# TNBC response to paclitaxel phenocopies interferon response which reveals cell cycle-associated resistance mechanisms

**DOI:** 10.1038/s41598-024-82218-9

**Published:** 2025-02-04

**Authors:** Nicholas L. Calistri, Tiera A. Liby, Zhi Hu, Hongmei Zhang, Mark A. Dane, Sean M. Gross, Laura M. Heiser

**Affiliations:** 1 Biomedical Engineering Department, Oregon Health & Science University, Portland, OR USA; 2 Knight Cancer Institute, Oregon Health & Science University, Portland, OR USA

**Keywords:** Triple negative breast cancer (TNBC), Single-cell RNA sequencing (scRNA-seq), Transcription factor, Cell cycle, Interferon response, Live-cell imaging, Breast cancer, Cytokinesis, Gene regulatory networks

## Abstract

Paclitaxel is a standard of care neoadjuvant therapy for patients with triple negative breast cancer (TNBC); however, it shows limited benefit for locally advanced or metastatic disease. Here we used a coordinated experimental-computational approach to explore the influence of paclitaxel on the cellular and molecular responses of TNBC cells. We found that escalating doses of paclitaxel resulted in multinucleation, promotion of senescence, and initiation of DNA damage induced apoptosis. Single-cell RNA sequencing (scRNA-seq) of TNBC cells after paclitaxel treatment revealed upregulation of innate immune programs canonically associated with interferon response and downregulation of cell cycle progression programs. Systematic exploration of transcriptional responses to paclitaxel and cancer-associated microenvironmental factors revealed common gene programs induced by paclitaxel, IFNB, and IFNG. Transcription factor (TF) enrichment analysis identified 13 TFs that were both enriched based on activity of downstream targets and also significantly upregulated after paclitaxel treatment. Functional assessment with siRNA knockdown confirmed that the TFs FOSL1, NFE2L2 and ELF3 mediate cellular proliferation and also regulate nuclear structure. We further explored the influence of these TFs on paclitaxel-induced cell cycle behavior via live cell imaging, which revealed altered progression rates through G1, S/G2 and M phases. We found that ELF3 knockdown synergized with paclitaxel treatment to lock cells in a G1 state and prevent cell cycle progression. Analysis of publicly available breast cancer patient data showed that high ELF3 expression was associated with poor prognosis and enrichment in programs associated with cell cycle progression. Together these analyses disentangle the diverse aspects of paclitaxel response and identify ELF3 upregulation as a putative biomarker of paclitaxel resistance in TNBC.

## Introduction

Triple negative breast cancer (TNBC) is an aggressive form of breast cancer that affects 10–20% of all breast cancer patients and is characterized by its lack of expression of estrogen, progesterone and HER2 receptors^1^. The standard of care for TNBC patients primarily relies on conventional anthracycline and taxane-based chemotherapy regimens, and few next-generation therapies have shown efficacy in patients with this disease^2^. Paclitaxel, a taxane-based chemotherapeutic commonly used in TNBC treatment^3^, targets microtubules to disrupt the formation of the mitotic spindle, resulting in cell cycle arrest and apoptosis. Paclitaxel treated cells often fail to achieve symmetric division of nuclear content, potentially resulting in apoptosis^4^. Cells that persist through paclitaxel treatment may exhibit nuclear damage in the form of either disrupted nuclear structures and/or acquired polyploidy^5,6^. While 22% of TNBC patients treated with paclitaxel achieve pathological complete response, the outcome for those with residual disease is relatively poor^7,8^. Moreover, paclitaxel monotherapy only achieves a median 5.5 month progression free survival in patients with locally advanced or metastatic disease^9^. Therefore, there is a need to better understand the molecular basis of paclitaxel response and mechanisms of resistance that may be targeted for therapeutic benefit.

Phenotypic plasticity enables malignant cells to rapidly adapt to therapeutic challenge^10^ and can also drive acquired drug resistance^11^. Adaptive responses often involve activation of new transcription factors that in turn upregulate programs that repress immune activation^12^, grant tolerance to DNA replication stress^13^, or enable evasion of apoptosis ^14^. Single-cell RNA sequencing (scRNA-seq) is a powerful approach to investigate the subtle but critical differences in transcriptional landscape that distinguish cellular phenotypic states and to identify molecular programs associated with different therapeutic sensitivities^15–17^.

To elucidate adaptive responses of TNBC cells to paclitaxel, we performed deep single-cell RNA sequencing of HCC1143 TNBC cells before and after paclitaxel treatment. Paclitaxel induced a range of phenotypic changes, including altered cell cycle phase distribution, increased proportion of multinucleated cells, increased expression of senescence and DNA damage associated biomarkers, and upregulation of interferon-related gene programs. Comparison of gene expression profiles from paclitaxel treated versus IFNB or IFNG treated cells enabled identification of genes that were uniquely upregulated after paclitaxel treatment, including a suite of transcription factors. Functional assessment with siRNA knockdown confirmed that many of these TFs are critical for mediating resistance to paclitaxel. Using live-cell imaging, we probed the temporal dynamics of these functional responses, which demonstrated that knockdown of ELF3, FOSL1 and IRF9 synergize with paclitaxel to slow cell cycle progression. Together, these analyses identify upregulation of ELF3, FOSL1 and IRF9 as important regulators of cell cycle progression that mediate paclitaxel response, and which may serve as biomarkers of response.

## Results

### Paclitaxel modulates multiple cancer-associated phenotypes

**Fig. 1:**
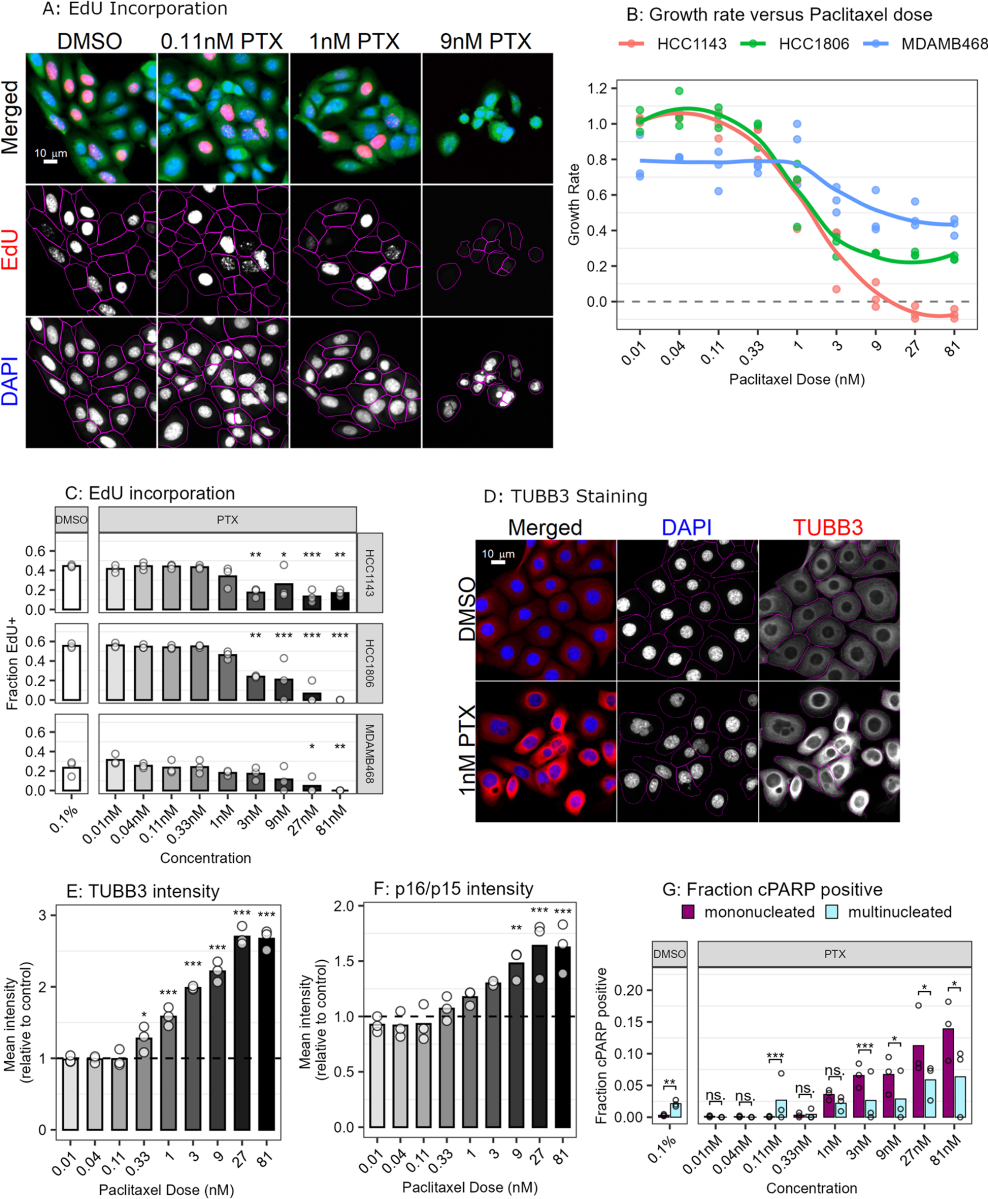
Paclitaxel modulates multiple cancer associated phenotypes. (**A**) Representative fluorescent images showing EdU incorporation for HCC1143 cells treated with DMSO or Paclitaxel at the indicated doses for 72 h. Merged image shows DAPI (blue channel, marker for DNA), EdU (red channel, marker for active DNA synthesis) and HCS Cellmask (green channel, marker used as cytoplasmic segmentation aid). Monochrome images below show DAPI and EdU channels separately, and magenta outline indicates cytoplasmic boundary from segmentation. (**B**) Growth rates calculated for 72 h paclitaxel treatment for three TNBC cell lines: HCC1143, HCC1806, MDA-MB-468. Dots indicate individual measurements, line indicates loess fit of the points. Growth rate was calculated from phase confluency using an Incucyte S3. (**C**) Barplot indicating proportion of EdU + cells for each cell line at each paclitaxel concentration. Significance assessed with Dunnett’s test. (**D**) Representative fluorescent images showing HCC1143 cells treated with either 0.1% DMSO or 1 nM Paclitaxel for 72 h and then stained with DAPI (blue) and TUBB3 (red). Monochrome images show DAPI and TUBB3 separately, where magenta outline indicates cytoplasmic segmentation boundary. (**E–F**) Barplots showing mean TUBB3 (**E**) and p16/p15 (**F**) staining intensity for triplicate wells of HCC1143 treated with Paclitaxel dose escalation, after normalization to paired DMSO control (horizontal line). Mean intensity was calculated for the entire cell segmentation area (including nuclear and cytoplasmic regions). Significance assessed with Dunnett’s test. (**G**) Barplot comparing the fraction of cPARP positive cells for mononucleated (magenta) versus multinucleated (cyan) cells within the same treatment condition. cPARP positive threshold was set to the 99th quantile of DMSO treated cells total cPARP nuclear intensity (Supplemental Fig. 1C). Significance assessed with proportions test. For all statistics: * = *p* < 0.05, ** = *p* < 0.01, *** = *p* < 0.001.

We identified changes in growth and proliferation induced by paclitaxel by performing an EdU incorporation assay for three TNBC cell lines (HCC1143, HCC1806 and MDA-MB-468) treated for 72 h with dose escalation of paclitaxel (0.01 nM-81 nM). Higher paclitaxel doses (1 nM-81 nM) induced increasingly frequent nuclear aberrations, including multi-lobular and multinucleated phenotypes (Fig. 1A). Comparison of cell counts at 72 h to 0 h and DMSO control revealed a sigmoidal relationship between paclitaxel dose and growth rate for all three cell lines (Fig. 1B), where the highest concentrations were cytostatic (growth rate plateau >  = 0) for two of the three cell lines^18^. The total DAPI intensity and total EdU intensity for each cell was calculated and thresholds were used to classify cells into 2N/EdU-, EdU + or 4N/EdU- cell cycle states (Supplemental Fig. 1A). There was a significant reduction in the fraction cells staining positive for EdU (Fig. 1C), indicating that paclitaxel treatment reduces the proportion of cells that are actively synthesizing new DNA, as expected^6,19^.

We further assessed adaptive cellular responses by analyzing biomarkers associated with senescence (p16/p15), DNA damage induced apoptosis (cPARP), and microtubule components (TUBB3) for HCC1143 cells treated with paclitaxel dose escalation for 72 h (Fig. 1D). TUBB3 overexpression has been associated with resistance to multiple microtubule targeting drugs, and consistent with this, we found a dose-dependent relationship between TUBB3 expression and paclitaxel concentration (Fig. 1E)^20,21^. There was also a positive association between both cytoplasmic and nuclear p16/p15 staining and paclitaxel dose (Fig. 1F). Additionally, we observed a strong correlation between p16/p15 and TUBB3 expression at the single cell level across paclitaxel concentrations, suggesting that the TUBB3 highly expressing cells represent a senescent subpopulation of cells (Supplemental Figs. 1B,C, Pearson correlation = 0.70, r^2 = 0.5). Increasing doses of paclitaxel induced a corresponding increase in the fraction of cPARP positive cells (DMSO: 6%, 81 nM Paclitaxel: 28% cPARP positive), indicating induction of DNA damage driven apoptosis. Higher paclitaxel doses resulted in a significantly higher proportion of mononucleated cells staining positive for cPARP as compared to multinucleated (19.4% mononucleated cells and 7.1% multinucleated cells cPARP positive at 81 nM paclitaxel, proportions test *p* = 0.017), suggesting that multinucleated cells are less likely to undergo DNA damage-induced apoptosis (Fig. 1G). Comparison between these findings and the EdU results showed that across a range of paclitaxel doses, multinucleated cells tended to belong to the 4N/EdU- cell cycle state (Supplemental Fig. 1D). Together this suggests that multinucleated cells that survive paclitaxel treatment are cell cycle arrested and less likely to undergo DNA damage-induced apoptosis than are mononucleated cells.

### Cells surviving paclitaxel treatment halt cycling and upregulate interferon response genes

**Fig. 2:**
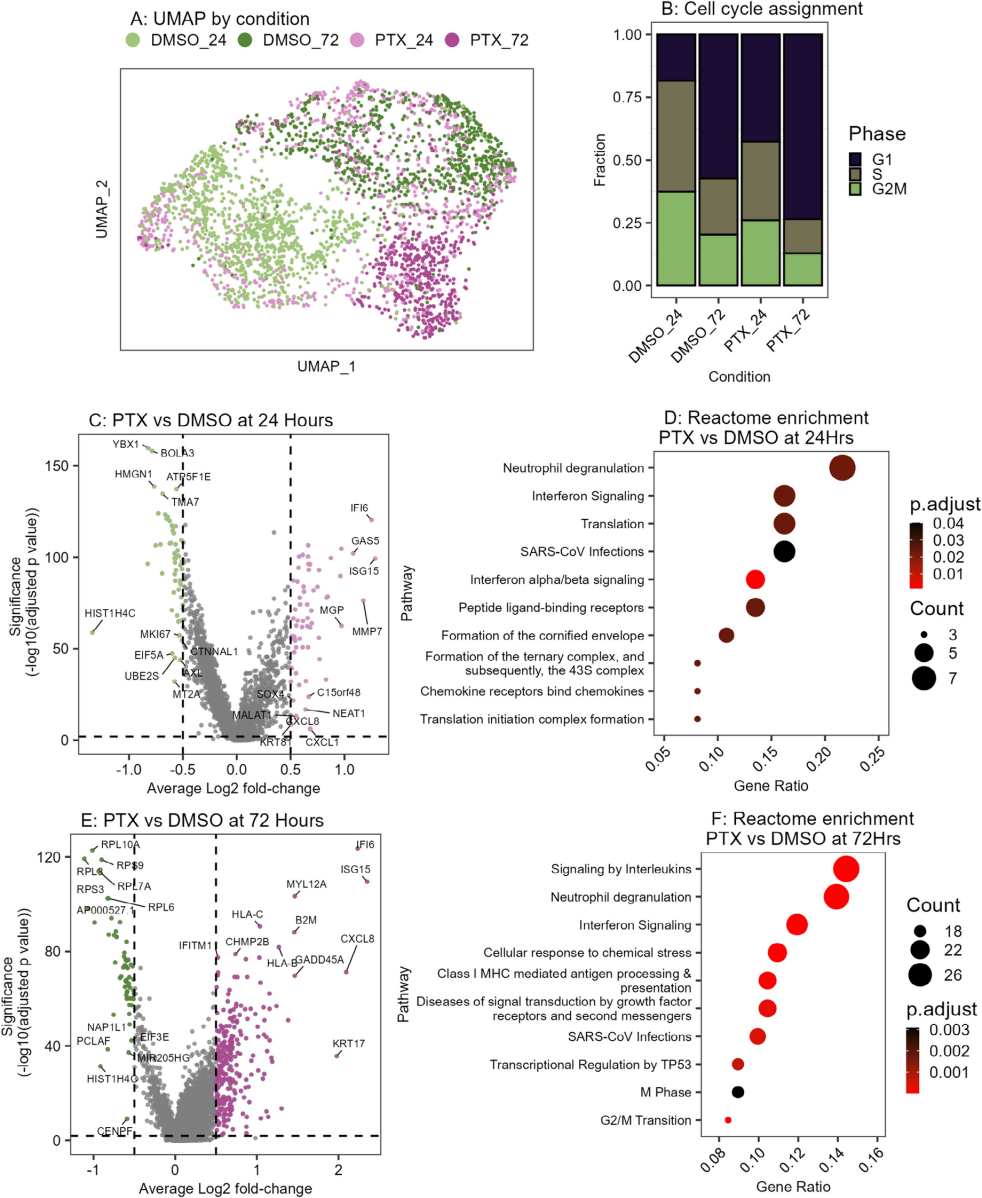
Cells surviving paclitaxel treatment have halted cycling and upregulated interferon response genes. (**A**) UMAP color coded by treatment condition. DMSO_24 = 0.1% DMSO for 24 h, DMSO_72 = 0.1% DMSO for 72 h, PTX_24 = 1 nM Paclitaxel for 24 h, PTX_72 = 1 nM Paclitaxel for 72 h. (**B**) Barplot showing proportion of each condition assigned to G1, S, or G2M cell cycle state based on transcriptional profile. (**C**) Volcano plot of differentially expressed genes for Paclitaxel treatment versus DMSO at 24 h. (**E**) Significantly differentially expressed genes were determined with cutoffs of Benjamini Hochberg corrected *p* < 0.05 and absolute Log2FoldChange > 0.5. (**D**) Reactome pathway enrichment results for genes significantly upregulated after paclitaxel treatment at 24 h. Size indicates the number of genes upregulated within the pathway, color indicates significance. (**E**) Volcano plot of differentially expressed genes for Paclitaxel treatment versus DMSO at 72 h. Differentially expressed genes determined as in (**C**). (**F**) Reactome pathway enrichment results for genes significantly upregulated after paclitaxel treatment at 72 h. Size indicates the number of genes upregulated within the pathway, color indicates significance.

We assessed paclitaxel-induced molecular programs with 10X Genomics single-cell whole transcriptome sequencing of HCC1143 cells treated with either DMSO vehicle control or 1 nM paclitaxel for 24 h or 72 h (Fig. 2A). After quality control filtering that required cells to have a minimum of 3000 unique genes and a maximum of 25% mitochondrial counts, we recovered 3194 total cells (513 – 1106 cells per condition) with a mean UMI count of 63,668 (Supplemental Fig. 2A).

We examined drug-induced changes in cell cycle distribution by assigning cell cycle status to each individual cell using aggregate expression of canonical gene programs for S and G2/M^18,19^. In agreement with the results of the EdU incorporation assay (Fig. 1C), we observed a reduction in the fraction of S phase cells after paclitaxel treatment as compared to time-matched vehicle control (Fig. 2B). UMAP visualization showed that the cells organized overall by treatment condition and cell cycle phase (Supplemental Fig. 2B).

We analyzed time-matched conditions to identify significantly differentially expressed genes induced by paclitaxel treatment (Wilcoxon rank sum test, absolute log2 fold-change > 0.5, Benjamini Hochberg FDR < 0.01). This revealed a time-dependent change in molecular programs with 66 significantly upregulated and 57 significantly downregulated genes after 24 h of paclitaxel treatment, and 256 significantly upregulated genes and 58 significantly downregulated genes after 72 h (Fig. 2C, Supplemental Fig. 2C). Reactome pathway enrichment analysis revealed that the significantly upregulated genes from the 24 h paclitaxel treated sample were enriched for multiple programs related to Interferon Signaling and Translation (Fig. 2D, Supplemental Data 2). Programs uniquely upregulated after 72 h of paclitaxel treatment include Response to Chemical Stress, Cell Cycle Progression, and Antigen Processing-Cross presentation (Fig. 2E-F). The ontologies enriched after 72-h paclitaxel treatment had low overlap with those at 24 h, suggesting that paclitaxel response is a dynamic process with transcriptional changes that continue to emerge even after the first 24 h of treatment (Jaccard Index = 0.023, Supplemental Fig. 2D). Notably, the Neutrophil Degranulation pathway was significantly enriched at both time points, with upregulated genes related to antigen presentation (HLA-B, HLA-C, B2M) and differentiation (CD47, CD55, CD59, CD63). Paclitaxel treatment also induced significant upregulation of the pro-tumorigenic chemokines CXCL1 and CXCL8 (Supplemental Fig. 2E)^22–25^. Together this shows that TNBC cells that survive paclitaxel treatment have altered surface marker expression and produce tumor supportive chemokines.

### Paclitaxel response activates canonical interferon response genes

Despite gene enrichment consistent with interferon response, the paclitaxel treated cells showed no evidence of autocrine interferon signaling, indicating that paclitaxel induces interferon response pathways in a non-canonical manner (Supplemental Fig. 3A). To disentangle the paclitaxel response signature from an interferon-induced response, we performed a second scRNA-seq experiment with HCC1143 cells after treatment for 72 h with 7 perturbations that target ligand-receptor pairs known to play an important role in normal and pathological breast tissue^26,27^: Interferon Beta (IFNB), Interferon Gamma (IFNG), Transforming Growth Factor Beta (TGFB), Oncostatin M (OSM), Lymphotoxin Alpha (LTA), Notch Inhibitor (NOTCHi) and combination of Notch Inhibitor and Interferon Beta (NOTCHi_IFNB). Cells were treated for 72 h and then harvested and sequenced with the 10X Genomics scRNA-seq pipeline. After quality control filtering, we recovered 4231 total cells (295–725 cells per condition, Supplemental Fig. 3B).

**Fig. 3:**
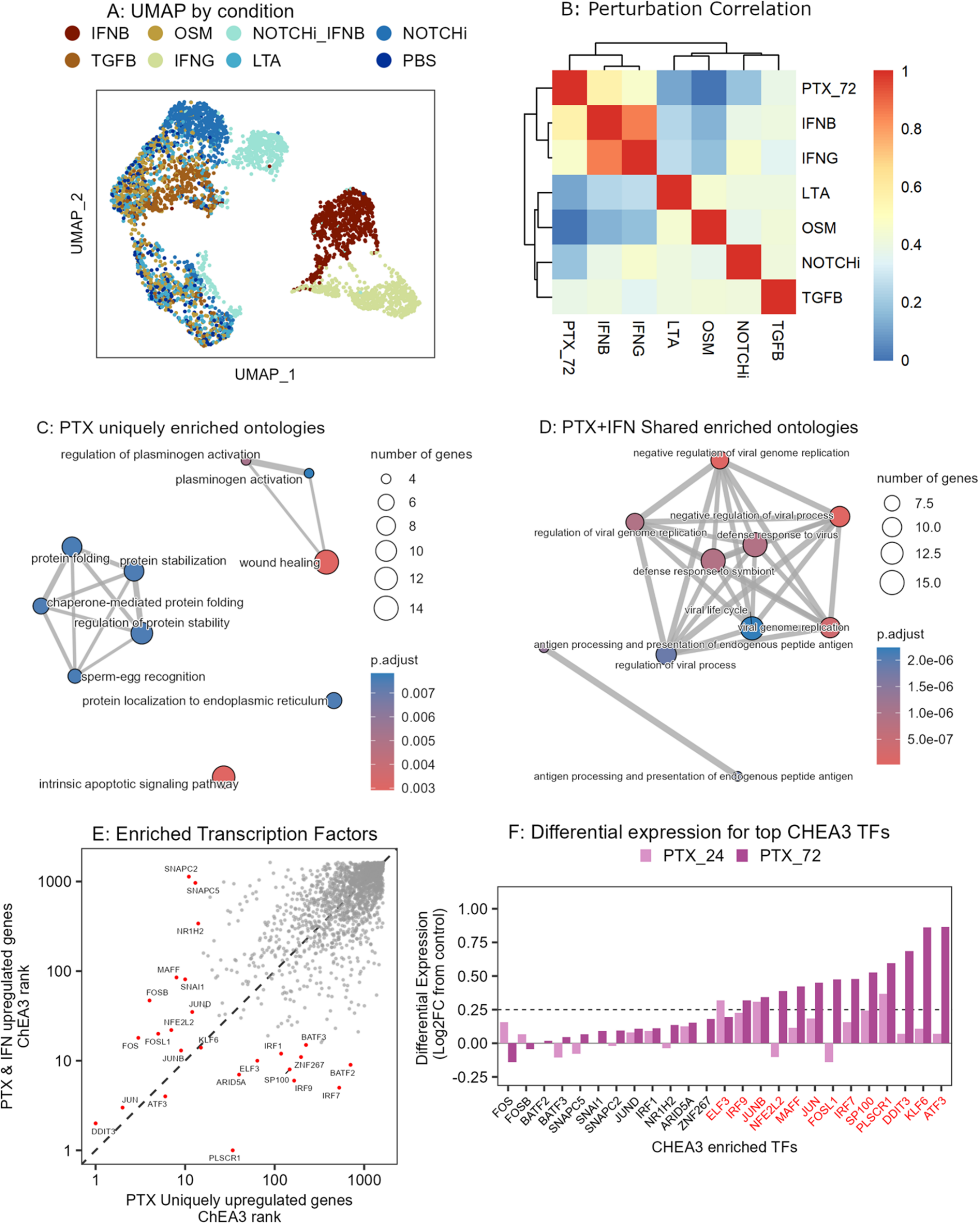
Paclitaxel response activates canonical interferon response genes. (**A**) UMAP showing the scRNA-seq landscape after ligand perturbations. IFNB = Interferon-Beta, OSM = Oncostatin-M, NOTCHi_IFNB = Notch inhibitor + Interferon-Beta, NOTCHi = Notch inhibitor, TGFB = Transforming Growth Factor Beta, IFNG = Interferon-Gamma, LTA = Lymphotoxin-Alpha, PBS = Phosphate Buffered Saline (control). (**B**) Heatmap showing the Pearson correlation for log2 fold-change values for each perturbation versus time-matched control. (**C**,**D**) Gene enrichment map for Paclitaxel uniquely upregulated (3C) and Paclitaxel + Interferon shared upregulated (3D) genes. Color indicates significance, size indicates number of upregulated genes, and lines connect ontologies with shared elements. (**E**) ChEA3 transcription factor enrichment ranks computed from 140 Paclitaxel uniquely upregulated genes (x axis) versus 120 Paclitaxel-Interferon shared upregulated genes (y axis). Lower rank indicates higher imputed activity, and named TFs with red dots were within the top 15 ranks for either geneset. TFs to the lower right of the diagonal have higher imputed activity within the PTX + IFN shared upregulated gene set, and TFs to the upper left of the diagonal have higher imputed activity within the PTX uniquely upregulated gene set. (**F**) Bar plot showing Average Log2FC from paclitaxel treated scRNA-seq data for the 24 top ranked transcription factors (intersect of top 15 ranked for PTX unique or PTX shared individually). Red font indicates TFs significantly upregulated (average log2 fold-change > 0.25, FDR < 0.01) at either 24 or 72 h of paclitaxel treatment compared to vehicle control.

UMAP projection of scRNAseq profiles revealed that ligand-treated cells largely grouped by perturbation (Fig. 3A) and cell cycle state (Supplemental Fig. 3C). The IFNB, IFNG, TGFB, NOTCHi and NOTCHi_IFNB conditions all had an increase in proportion of G1 cells compared to control, suggesting these ligands are cytostatic in this cell line (Supplemental Fig. 3D). Based on the observation that paclitaxel induced interferon related pathways, we next sought to evaluate the similarity in transcriptional response between paclitaxel and ligand perturbations. To that end, we computed the differential expression of all genes for each perturbation compared to time-matched vehicle control and then evaluated the pairwise Pearson correlation of log2 fold-change values (Fig. 3B). The IFNB and IFNG conditions were the most strongly correlated (Pearson correlation = 0.86), indicating a conserved impact on transcription despite acting through different receptors. The 72 h paclitaxel condition was highly correlated with both interferon treatments (IFNB Pearson correlation = 0.57, IFNG Pearson correlation = 0.47) as compared to the other single-agent perturbations (0.0, 0.11, 0.18, 0.38 Pearson correlation with OSM, LTA, NOTCHi and TGFB, respectively). Despite the strong transcriptional overlap between interferon treated and paclitaxel treated samples, we observed no multinucleation following interferon treatment (Supplemental Fig. 3E).

While type 1 and type 2 interferons primarily exhibit antitumor effects through activation of the immune system, some studies have shown that they have direct effects through induction of cell cycle arrest or apoptosis in malignant cells^28,29^. To further clarify the overlapping transcriptional responses of paclitaxel and interferon, we next sought to differentiate between pathways that were uniquely induced by paclitaxel response or that represent common responses induced by paclitaxel or interferon. Reactome pathway enrichment analysis revealed that the 140 genes upregulated after paclitaxel treatment but not after IFNG or IFNB (“paclitaxel-unique”) were enriched in molecular programs related to wound healing, protein folding, and intrinsic apoptotic signaling pathway (Fig. 3C), whereas the 117 genes upregulated by all three treatments (“paclitaxel-shared”) were associated with defense response to virus and antigen presentation (Fig. 3D).

As paclitaxel has been shown to activate cGAS/STING and subsequently induce upregulation of Interferon related genes in human breast cancer, we hypothesized that the strong overlap in interferon and paclitaxel transcriptional responses was driven by a shared increase in transcription factor (TF) activity^5,30,31^. We identified enriched TFs in our paclitaxel-unique and paclitaxel-shared gene signatures using ChEA3^32^, which evaluates the expression of gene targets downstream from a TF of interest. DDIT3, JUN, KLF6, and ATF3 emerged as enriched transcription factors across both the paclitaxel-unique and paclitaxel-shared gene signatures (Fig. 3E). The paclitaxel-unique genes were enriched for TFs in the Immediate-Early Gene family, including JUN (JUN, JUNB, JUND) and FOS (FOS, FOSL1, FOSB)^33^. TFs enriched from the shared gene list were associated with activity of Interferon Regulatory Factors (IRF1/IRF7/IRF9) and Basic Leucine Zipper family (BATF2/BATF3), both related to antiviral response and regulation of antigen-presenting cells^34–36^. The high activity of IRF7 is consistent with activation of the cytosolic nucleotide sensor RIGI, suggesting that the nuclear damage induced by paclitaxel drives an increase in cytosolic RNA or DNA ^37^.

### Inhibition of paclitaxel-induced transcription factors alters proliferation and nuclear morphology

We used siRNA knockdown in three basal-like TNBC cell lines (HCC1143, HCC1806, MDA-MB-468) to functionally assess prioritized TFs implicated in modulating response to paclitaxel. We nominated a panel of 13 TFs for functional testing, based on ChEA3 analysis and change in gene expression after 24 or 72 h of paclitaxel treatment (Fig. 3F, Log2FC > 0.25, Benjamini–Hochberg FDR < 0.01). The expression of the implicated TFs were not correlated at the single-cell level (Supplemental Fig. 3F), but several were upregulated after interferon treatment (Supplemental Fig. 4A). Most of the TFs included in this panel were either subunits of (ATF3, FOSL1, JUN, JUNB, MAFF)^38^ or known interactors with (ELF3, IRF7, DDIT3, NFE2L2)^39–42^ the AP-1 transcription factor family. Dysregulation of the AP-1 pathway is associated with multiple tumorigenic phenotypes including enhanced cellular growth, proliferation, and survival^43^. To functionally assess the role of these TFs in paclitaxel response, cells were transfected with siRNA for 24 h, then treated for 72 h with paclitaxel or DMSO, and subsequently fixed and stained with DAPI (nuclear marker). The resultant images were subjected to quantitative image analysis to identify nuclear and cellular masks, followed by assessment of total cell number and fraction of multinucleated cells for each condition.

**Fig. 4:**
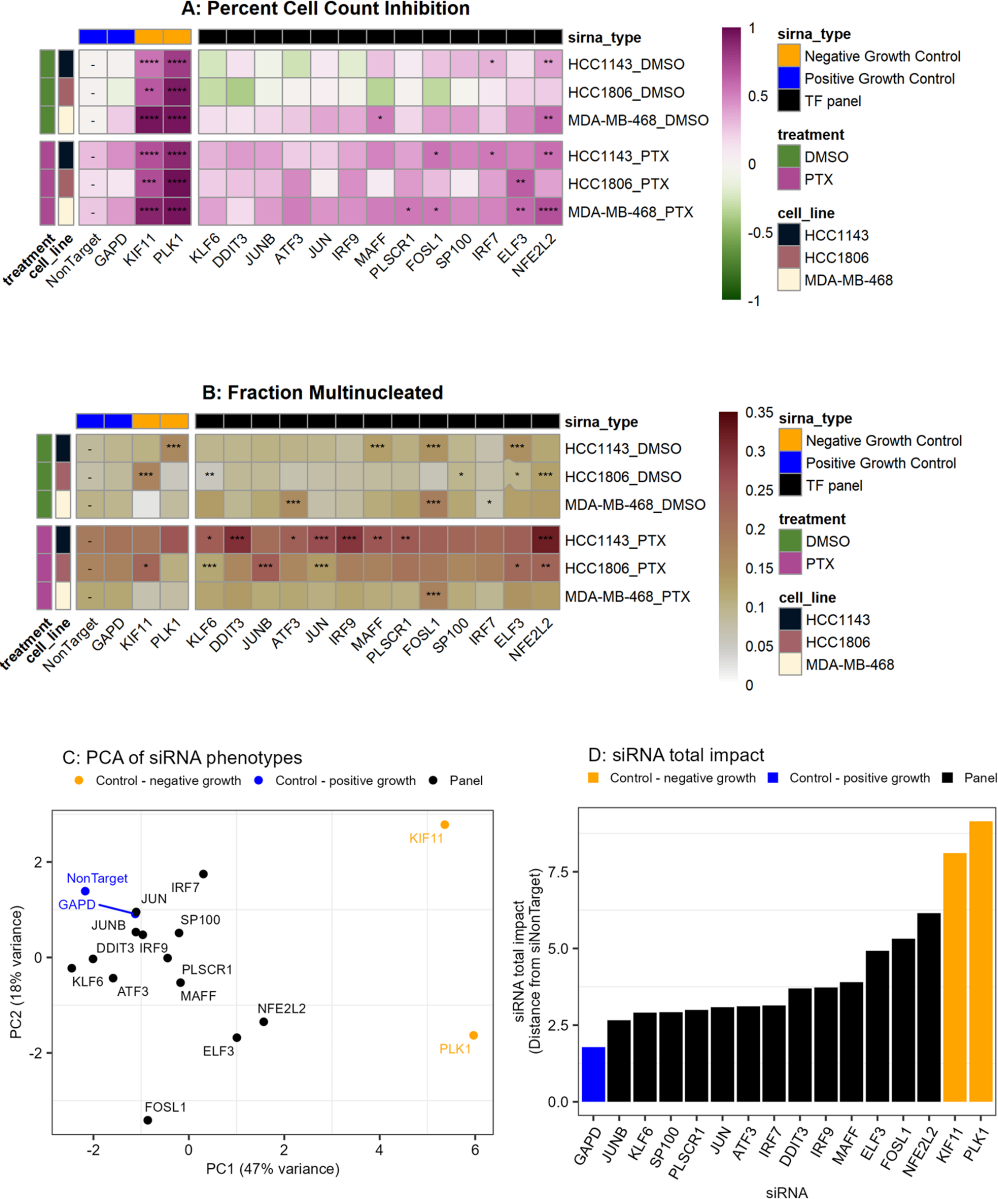
Inhibition of paclitaxel-induced transcription factors alters proliferation and nuclear morphology. (**A-B**) Heatmaps showing percent inhibition of cell count (A) and proportion of multinucleated cells (B). Cell count is normalized to the same cell line DMSO + siNonTarget control, and color indicates mean value across triplicate. Significance for percent inhibition computed with two-tailed Student’s T-test with Bonferroni correction, and significance for fraction multinucleated computed with proportions test with Bonferroni correction. siNonTarget and siGAPD represent positive growth controls, and siPLK1 and siKIF11 represent negative growth controls. (**C**) Principal Component Analysis results for each siRNA knockdown where each combination of cell line (HCC1143, HCC1806, MDA-MB-468), feature (relative cell count, fraction multinucleated) and condition (DMSO, PTX) is considered a feature. (**D**) The Euclidean feature-distance from NonTarget control for each siRNA. For all statistics: * = *p* < 0.05, ** = *p* < 0.01, *** = *p* < 0.001.

First, we analyzed the influence of TF knock-down on cell count after 72 h to evaluate their effects on cell viability. We found that knockdown of 3 of 13 TFs (NFE2L2, IRF7, MAFF) in the absence of paclitaxel significantly reduced cell numbers for at least one cell line (Student’s t-test, *p* < 0.05, Fig. 4A, upper). We then examined the influence of TF knock-down in the presence of paclitaxel to test our hypothesis that upregulation of these TFs mediates adaptive resistance. Knockdown of 5 of 13 TFs (NFE2L2, ELF3, IRF7, FOSL1, PLSCR1) in combination with paclitaxel significantly lowered cell count in at least one cell line compared to paclitaxel alone (Student’s t-test, *p* < 0.05, Fig. 4A, lower).

We hypothesized that these 13 TFs may also be involved in cytokinesis, based the changes we observed in nuclear morphology following paclitaxel treatment (Fig. 1A). We found that siRNA knockdown alone caused a significant increase in the fraction of multinucleated cells for 6 of 13 TFs for at least one cell line: NFE2L2, ELF3, SP100, FOSL1, MAFF, and ATF3 (Proportions test, *p* < 0.05. Figure 4B). Additionally, knockdown for 11 of 13 TFs in the presence of paclitaxel resulted in significantly increased fraction of multinucleated cells as compared to paclitaxel alone, for at least one cell line. These findings suggest an important role for these transcription factors in maintaining nuclear structure and achieving symmetric cytokinesis in proliferating breast cancer cells.

We next sought to identify which transcription factors within this panel induced the largest overall impact on cell phenotype across treatments and cell lines. Here, we considered each siRNA an independent sample and each combination of cell line (HCC1143, HCC1806, MDA-MB-468), treatment (DMSO, paclitaxel) and phenotype (relative cell count, fraction multinucleated) as 12 independent features. Principal component analysis applied to the transformed data revealed that our positive and negative growth controls separated along Component 1 (Fig. 4C). To identify the TFs that had the greatest overall impact on phenotype, we computed the Euclidean feature-distance (distance for z-scored features) to identify TFs that induced the greatest feature-distance from siNonTarget positive growth control. We found that ELF3, FOSL1, and NFE2L2 knockdown had the largest Euclidean feature-distance, indicating that knockdown had a strong effect on both proliferation and regulation of nuclear morphology across the three TNBC cell lines (Fig. 4D). Protein quantification for ELF3, FOSL1 and NFE2L2 confirmed that each siRNA was effective at reducing protein levels for the target transcript (Supplemental Figs. 4A-B).

### ELF3 mediates cell cycle progression under paclitaxel treatment

**Fig. 5:**
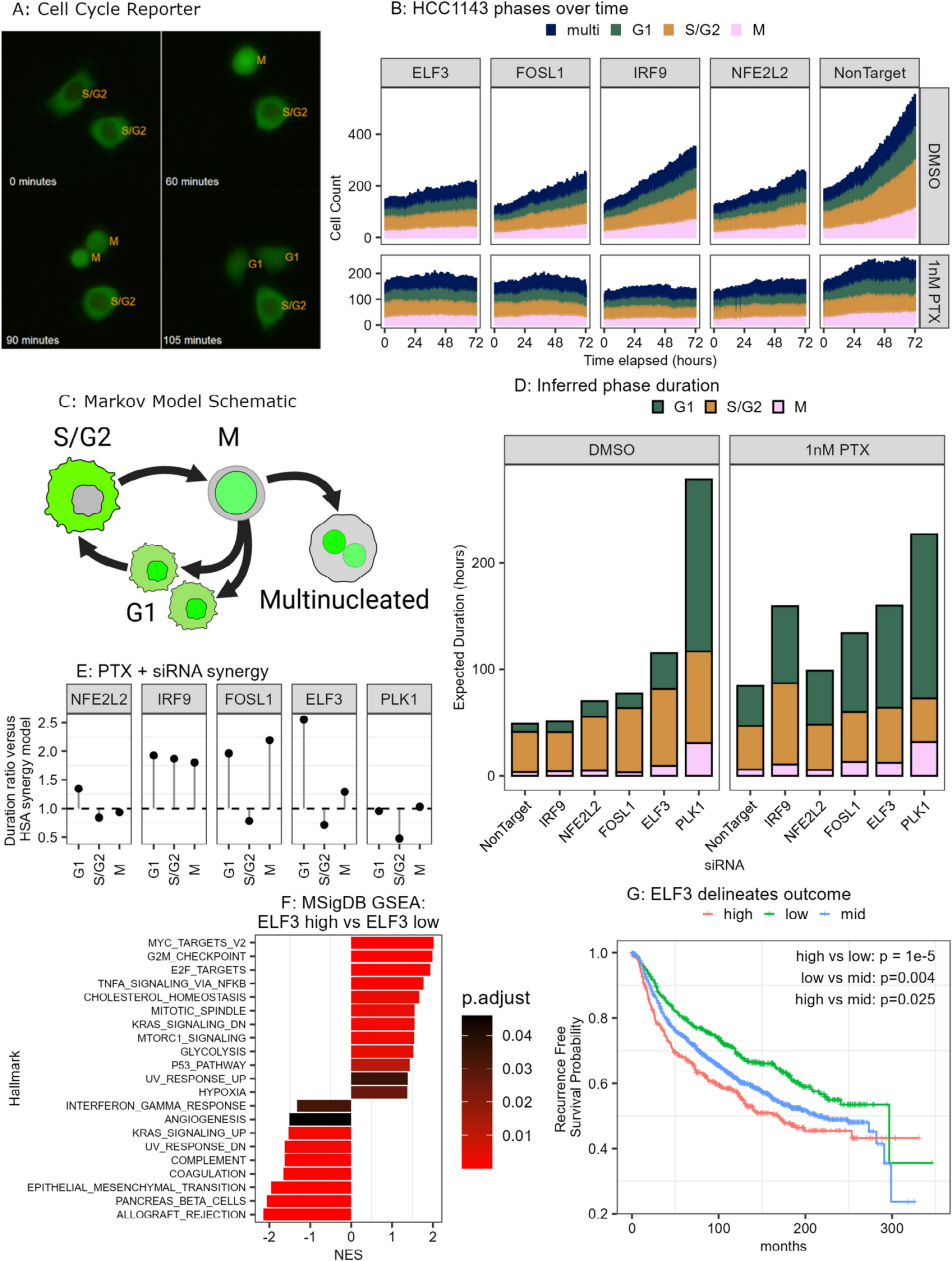
ELF3 mediates cell cycle progression under paclitaxel treatment. (**A**) Representative images showing the HCC1143 cell cycle reporter line and a mitotic even occurring over 105 min. Orange text indicates assigned cell cycle. (**B**) Relative cell count for each cell cycle phase over time for each siRNA condition + /− paclitaxel (PTX). Cell counts normalized to total cell number at earliest time point. (**C**) Schematic showing the underlying structure of permitted transitions used in the Markov Model. (**D**) Barplot showing inferred cell cycle phase duration from Markov model transition rates for each treatment combination. (**E**) PTX + siRNA synergy computed as the ratio of inferred phase duration for combination (siRNA + PTX) versus Highest Single Agent (HSA, highest duration for either siRNA or PTX treatment alone). Value of 1 indicates no change in combination, values greater than 1 indicate synergy and values less than 1 indicate antagonism. (**F**) MSigDB Gene Set Enrichment (GSEA) results for ELF3 high versus ELF3 groups for samples from the METABRIC cohort. (**G**) Overall survival for the METABRIC breast cancer cohort stratified by ELF3 mRNA expression. High = top quartile of ELF3 expression, IQR = inner quartile range of ELF3 expression, and low = lowest quartile of ELF3 expression.

Motivated by the observation that many anti-cancer drugs act by targeting the cell cycle, we next explored the influence of prioritized TFs on cell cycle progression by leveraging HCC1143 cells genetically engineered to express a cell cycle reporter. The cell cycle state of HDHB-mClover/NLS-mCherry HCC1143 cells can be determined by quantification of relative HDHB-mClover (nuclear translocating cell cycle reporter) intensity within the cytoplasm compared to the nuclear signal marked by NLS-mCherry (stable nuclear localization)^44,45^. Cells in G1 cell cycle phase have near-equal nuclear and cytoplasmic HDHB-mClover expression, cells in S/G2 cell cycle phases exclude the HDHB-mClover from the nucleus, and cells in M phase concentrate the HDHB-mClover expression to the nucleus. Here we focused on NFE2L2, ELF3 and FOSL1, which induced the largest phenotypic effects; we additionally tested IRF9 which has been previously linked to anti-microtubule chemotherapy resistance ^46^. Reporter cells were subjected to siRNA transfection for 24 h and then treated with either 1 nM paclitaxel or DMSO. Treated cells were imaged every 15 min for 72 h. Nuclear and cytoplasmic masks were segmented with custom trained Cellpose^47^ models and the resultant data used to classify cells into four phase assignments based on HDHB-mClover expression and their number of nuclei (Fig. 5A). Mononucleated cells were assigned as G, S/G2 or M phase and multinucleated cells assigned to either M or multinucleated phase based on localization of the HDHB-mClover signal (Supplemental Fig. 5A).

As chemotherapeutic drugs often have peak efficacy during a specific cell cycle phase, we next asked whether the combination of paclitaxel treatment and siRNA knockdown altered the dynamics of cell cycle progression. To that end, we trained a Markov Model on the live-cell data, which enabled us to infer transition rates and the average time spent in each of the four phases for a given treatment condition^44,48^. This approach uses the change in fraction of cells in each cell cycle phase over time (Fig. 5B) to learn cell cycle-specific transition rates, which represent the fraction of cells that transition from one phase to another phase within a 1 hour timestep (Supplemental Fig. 5B). We constrained our model such that proliferating cells can either successfully complete the cell cycle or undergo mitotic failure into a permanent multinucleated phase (Fig. 5C). The resultant model can reconstruct the cell phase distribution and counts for the entire experimental duration using just the data from the initial timepoint and learned transition rates (Supplemental Figs. 5C,D). The model-predicted values for both siRNA and siRNA + Paclitaxel conditions had low error (Supplemental Fig. 6A,B), and the majority of the model predictions (11 of 12) had over 95% agreement with observed counts across all timepoints (Supplemental Fig. 6C).

We leveraged this model to assess how the combination of siRNA knockdown with paclitaxel synergized to disrupt the cell cycle and modulate transitions between phases. For each individual or combination perturbation, we computed the inferred phase duration for G1, S/G2 and M phases using the model’s homotypic transition rates, which represent the fraction of cells that remain in the same phase through the timestep (Fig. 5D). The inhibition of ELF3 alone strongly increased cell cycle duration (DMSO + siNonTarget = 49 h, DMSO + siELF3 = 115 h), with substantial increases to the time spent in G1 (DMSO + siNonTarget = 7.9 h, DMSO + siELF3 = 33.5 h) and S/G2 (DMSO + siNonTarget = 37.7 h, DMSO + siELF3 = 72.1 h) phases. The combination of paclitaxel treatment and siRNA knockdown resulted in the longest cell cycle durations for both the PTX + siELF3 (160 h) and PTX + siIRF9 (159 h) conditions compared to PTX + siNonTarget (84.4 h). To further assess the therapeutic impact of siRNA knockdown and paclitaxel combination treatments, we used a Highest Single Agent (HSA) model to compare the inferred phase duration under combination paclitaxel + siRNA to the highest inferred phase duration for a single agent (either paclitaxel alone or siRNA alone, Fig. 5E)^49^. In this approach, a duration ratio less than 1 indicates antagonism (siRNA + paclitaxel results in shorter inferred phase duration than highest single agent), a duration ratio of 1 means there is no benefit of combination compared to highest single agent, and a duration ratio greater than 1 indicates a positive synergistic effect (siRNA + paclitaxel results in longer inferred phase duration than highest single agent). Although knockdown of NFE2L2 alone resulted in longer cell cycle phases, these changes were not particularly synergistic with paclitaxel treatment. In contrast, IRF9 knockdown resulted in increased duration ratios of all three phases compared to HSA (G1: 1.93, S/G2: 1.87, M: 1.8), while FOSL1 knockdown resulted in increased duration ratios for G1 and M phases (1.96, 2.19 ratios respectively). ELF3 knockdown showed the greatest synergy for the G1 phase, with a G1 duration ratio of 2.55, indicating that the combination of ELF3 knockdown with paclitaxel treatment strongly inhibits cell cycle progression out of G1.

Motivated by these ELF3 observations, we hypothesized that ELF3 expression may be predictive of overall survival in breast cancer. To that end we assessed the METABRIC^50^ breast cancer cohort, using ELF3 expression to stratify patients into three categories: ‘high’ (Upper quartile of ELF3 expression), ‘mid’ (Inter quartile range of ELF3 expression), and ‘low’ (Lower quartile of ELF3 expression). We then compared the gene expression between the ELF3-high and ELF3-low groups and found that the ELF3-high tumors were significantly enriched for MSigDB hallmarks related to cell cycle progression (G2M_CHECKPOINT: NES = 1.99, Benjamini–Hochberg FDR = 5.0e−8, E2F_TARGETS: NES = 1.94, Benjamini–Hochberg FDR = 2.9e−7, Fig. 5F, Supplemental Fig. 7A). ELF3-low tumors were enriched for MSigDB hallmarks related to Allograft Rejection and Epithelial to Mesenchymal Transition (NES = -2.14, Benjamini–Hochberg FDR = 4.1e−11, and NES = − 1.95, Benjamini–Hochberg FDR = 6.1e−8 respectively). We next asked whether ELF3 expression levels were related to recurrence free survival in breast cancer patients, with the hypothesis that patients with high ELF3 expression would have worse survival. This comparison indicated that ELF3 high expressing tumors had significantly worse outcome than those with low ELF3 expression (HR = 1.57, *p* value = 1e−5, Fig. 5G). Additionally, this analysis found that ELF3 expression was prognostic in both directions when compared to the ELF3 mid-expressing group: ELF3-high tumors have significantly shorter recurrence free survival (HR = 1.21, *p* value = 0.025, Cox Proportional Hazard) and ELF3-low tumors have significantly longer overall survival (HR = 0.77, *p* value = 0.004, Cox Proportional Hazard) compared to the ELF3-mid tumors (Fig. 5G). These results support our experimental in vitro findings that ELF3 activity contributes to malignant cell proliferation, that high ELF3 expression is associated with cell cycle progression in human breast tumors, and finally that ELF3 may serve as a biomarker of progression free survival.

## Discussion

Paclitaxel is a cornerstone therapy for TNBC and is an important component of first line neoadjuvant treatment for newly detected disease. Despite this, less than 20% of breast cancer patients treated with combination neoadjuvant therapy achieve pathological complete response (pCR), and 47% of TNBC patients without pCR have recurrent disease within 10 years^51^. Although long-term chemotherapy resistance is often facilitated by clonal selection of growth-permissive mutations^52–54^, newer molecular profiling techniques have revealed that short-term adaptive responses emerge through rapid epigenetic changes without acquisition of new mutations^4,55^. In this study, we sought to identify adaptive responses that emerge after paclitaxel treatment and that may be targeted to deepen therapeutic response. To that end, we characterized the phenotypic and transcriptional responses of TNBC cells to paclitaxel, with a focus on changes in cell number, multinucleation, and transcription factor programs. Using siRNA knockdown, live-cell imaging, and computational modeling, we identified several TFs that phenocopied key aspects of paclitaxel response, including reduced proliferation rates and an increased proportion of multinucleated cells. ELF3 knockdown in vitro was synergistic with paclitaxel treatment and suppressed G1 to S/G2 cell cycle progression. Analysis of the METABRIC breast cancer cohort revealed that high expression of ELF3 was associated with worse outcome and higher cell-cycle related pathway activity. Together, these findings reveal a new role for ELF3 as a novel therapeutic target and biomarker of progression free survival for patients with TNBC.

Many prior drug and gene manipulation studies have focused on viability or other cell count proxies at a terminal timepoint^56–59^. While valuable, more recent studies have demonstrated that therapies modulate multiple cancer-associated hallmarks, including cell cycle phase behavior, senescence and nuclear morphology^44,60,61^. Further, cellular systems are inherently dynamic, and measures that capture temporal behavior are critical for gaining mechanistic insights^45,62–64^. While our live-cell studies captured important changes in cell cycle dynamics and the population distribution of various cell cycle states, no single metric captured the complete biological response. Future studies could deploy a richer panel of reporter molecules to gain deeper insights into the timing and order of transcription factor activation, activation of specific cell cycle checkpoints, or assess critical breast cancer pathways such as ERK, senescence or apoptosis^65^^66–68^.

In this study we identified dual roles of the transcription factor ELF3 that contribute to paclitaxel tolerance by: 1) permitting cells to transition from G1 to S/G2, and 2) enabling successful division into two mononuclear daughter cells. These findings were enabled by a Markov Model of cell cycle progression built on population level cell count data to learn the transition rates between cell cycle phases and inferred cell cycle phase durations^44,48^. While the inferred cell cycle durations represent an accurate prediction of the population’s average behavior, they cannot inform whether this arises from a homogenous or heterogenous distribution of cell cycle durations. This is of particular interest in the case of cancer treatment, as a small population of cells with a fitness advantage may eventually overtake other populations to achieve therapeutic resistance^69^. An alternative approach could track individual cells and their progeny to build complete lineages with accompanying cell cycle timing information. Lineage based approaches tend to be relatively low throughput due to the computational and experimental requirements, but they offer the opportunity to discern between heterogenous states of differing cycling speeds^48^. Another limitation of our Markov Model’s implementation is the assumption that transition rates are static throughout the observation duration. While the output of the model mapped well within the 72 h measurement window, there was some divergence at the end of the experiment that may suggest a weakening of either siRNA or paclitaxel effect. An updated model with temporally dynamic transition rates could provide additionally accuracy, enabling future studies aimed at prediction of drug combination effects and optimizing the drug schedule for maximum disruption of cell cycle progression. Optimal drug scheduling would be particularly interesting to examine, as TNBC disease is commonly treated with combination of paclitaxel with additional chemotherapies^3,7,51^.

Paclitaxel inhibits cell growth by simultaneously promoting microtubule assembly and inhibiting microtubule depolymerization, which results in mitotic checkpoint failure and subsequent apoptosis or senescent arrest^19,70^. The in vitro experimentation performed in this study represents an extensive investigation into the phenotypic and molecular responses of TNBC cells to paclitaxel, however we acknowledge that tumors are comprised of diverse cell types and microenvironmental signals not captured in this study^71^. Importantly, the tumor microenvironment has been shown to influence therapeutic response, including through cell–cell interactions^72–74^ and extracellular matrix remodeling^75,76^. In line with this, we observed that paclitaxel induced changes to malignant epithelial cells that suggest an altered capability to interact with immune cells via secretion of CXCL1 and CXCL8. Tumor-derived CXCL1 can recruit immunosuppressive myeloid cells that in turn inhibit CD8^+^ T cell infiltration^77^ while CXCL8 has been shown to recruit immunosuppressive neutrophils^78^, promote angiogenesis^79^ and maintain breast cancer stem cells^80^. Future studies that more deeply consider the influence of stromal and immune cell signals in modulating therapeutic response will be needed to create a complete picture of paclitaxel resistance.

As key regulators of multiple molecular programs, many transcription factors contribute to cancer-associated phenotypes^81^ and therapeutic response^82,83^. We found that the ETS family transcription factor ELF3 was upregulated during early response to paclitaxel treatment, and siRNA knockdown of ELF3 synergized with paclitaxel treatment to slow cell growth. Other studies have found that high ELF3 activity is associated with inhibition of epithelial to mesenchymal transition ^84^. Furthermore, ELF3 inhibition reduces proliferation across multiple cancer models including lung adenocarcinoma^85^, neuroendocrine carcinoma^86^ and prostate cancer^87^. Conserved dysregulation of ELF3 across cancer types may be related to its genomic location (loci 1q32) which is commonly amplified across cancers^89,90^ and also encodes for a number the cancer related genes including MDM4 (p53 suppressor)^91,92^. Our results support the idea that ELF3 is not only commonly dysregulated across these epithelial malignancies, but can also act as a transcriptional mechanism of acquired drug resistance. Future works that employ similar methodology to probe the function of ELF3 in these other malignancies, as well as systematically perturb combinations of the implicated transcription factors, may provide additional insight into this complex response phenotype and how it can be interrupted for therapeutic benefit.

Taken together, this work has identified ELF3 upregulation as an acquired mechanism of paclitaxel resistance. These findings support the development of pharmacological agents that inhibit ELF3 activity and could be used in combination with paclitaxel to further improve patient outcomes. While it has been historically difficult to develop targeted transcription factor inhibitors due to their lack of enzymatic activity, recent advances, such as targeted siRNA nanoparticles and indirect inhibition through targeting multiple interacting proteins, have made pharmacomodulation of transcription factors more tenable^93–95^. Until such therapies are developed, ELF3 may serve as a useful biomarker which predicts the development of paclitaxel resistance and continued malignant proliferation.

## Methods

### Cell culture

HCC1143 (ATCC), HCC1806 and MDA-MB-468 cells were authenticated by STR profiling and tested negative for mycoplasma. HCC1143 and HCC1806 cells were cultured in RPMI 1640 with L-glutamine (cat. 11875119, Life Technologies Inc.) supplemented with 10% fetal bovine serum (#16000-044, Gibco). MDA-MB-468 cells were cultured in DMEM (#11965-092, Life Technologies Inc.) supplemented with 10% fetal bovine serum (#16000-044, Gibco). All lines were incubated at 37C with 5% CO_2_. For perturbation experiments, cells were seeded into appropriate assay vessel for 24 h prior to treatment with either vehicle control (DMSO; PBS) or perturbation (table below).

**Table:** 

Perturbation	Shorthand	Concentration used for scRNA-seq	Source	Identifier	Vehicle
Paclitaxel	PTX	1 nM	LC labs	P-9600	0.1% DMSO
Notch inhibitor	NOTCHi	1 uM	Millipore sigma	BM0018-5MG	0.1% PBS
Interferon beta	IFNB	20 ng/mL	PBL assay science	11,410–2	0.1% PBS
Interferon gamma	IFNG	20 ng/mL	R&D systems	385-IR-100	0.1% PBS
Transforming growth factor beta	TGFB	10 ng/mL	Biotechne	7754BH005	0.1% PBS
Lymphotoxin	LT	10 ng/mL	Biotechne	8884-LY-025	0.1% PBS
Oncostatin M	OSM	10 ng/mL	Cell signaling technology	5367SC	0.1% PBS

### Fixed cell assays

Cells were plated at 3000 cells in 100ul of complete media per well in a 96 well plate (#08-772-225, FisherScientific). After 24 h, an additional 100ul of either vehicle (0.1% DMSO) or paclitaxel containing complete media was added. After 72 h cells were fixed with 4% Formaldehyde (#28908, ThermoFisher Scientific) for 15 min at room temperature, then permeabilized with 0.3% Triton X-100 (#X100-100ML, Sigma Aldrich) for 10 min at room temperature, then washed twice with PBS. Fixed cells were blocked with 1% BSA (A7906-100G, Millipore Sigma) in PBS for 1 h at room temperature and then stained overnight with 1:100 anti-CDKN2A/p16INK4A + CDKN2B/p15INK4B-AF644 (#ab199756, Abcam), and 1:100 anti-cPARP-AF647 (#6987S, Cell Signaling Technology) or 1:500 anti-TUBB3-AF647 (#ab190575, Abcam) overnight at 4C. Each well was washed twice with room temp PBS then stained with 0.5ug/mL DAPI (4083S, Cell Signaling Technology) in PBS for 15 min at room temperature. Following DAPI staining, wells were washed once with PBS, then stained with 1:20,000 HCS CellMask (Orange: #H32713, Green: #H32714, Invitrogen. Used for cytoplasmic segmentation) in PBS for 15 min at room temperature. Wells were washed twice with room temperature PBS and then 4 fields of view per well imaged on an InCell 6000 (GE Healthcare). Images were segmented with two custom Cellpose^47^ models to segment the nucleus (from DAPI channel) and cytoplasm (from HCS Cellmask channel). Image quantification was performed in R (v4.3.1) using EBImage (v4.42.0), and cells were annotated based on the number of distinct nuclei segmented within each cytoplasmic mask. EdU incorporation assays (#C10640, Invitrogen) were performed following manufacturer instruction with default concentrations, with an initial incorporation incubation time of 1 h at 37C, and additional staining with DAPI (#4083S, Cell Signaling Technology) and HCS CellMask (#H32714, Invitrogen) to aid in segmentation. Segmentation was performed using custom trained Cellpose models and feature quantification was performed with EBImage (v4.42.0).

### scRNA-seq library preparation and sequencing

Experiment 1 (DMSO 24 h, DMSO 72 h, Paclitaxel 24 h, Paclitaxel 72 h): Each condition had a single-cell RNA library prepared using 10X Genomics Single Cell 3’ v2 kits and sequenced on an Illumina NextSeq 500 for 500e6 reads per library.

Experiment 2: All conditions were multiplexed using Hashtag Oligonucleotide barcoding technology (TotalSeq-B, Biolegend) following manufacturer standard protocol. A paired feature-barcode library and mRNA library were generated using the Single Cell 3’ v3 kit (10X Genomics) following manufacturer instructions and then sequenced on an Illumina NovaSeq for 800e6 reads.

### scRNA-seq data processing

For both experiments; raw base call files were converted to FASTQ format with bcl2fastq (Illumina). Cellranger count (v6.0.2) was used to align reads to the GRCh38 transcriptome (GRCh38-2020-A, accessed from 10X Genomics) and count UMI reads. The R package Seurat^96,97^ (4.0.5) was used to perform variable feature identification, linear and nonlinear dimensionality reduction, unsupervised clustering and differential gene expression.

Variance Stabilizing Transformation was used to identify the top 2000 variable genes and Principal Component Analysis (PCA) was used to reduce these 2000 genes to 10 components for UMAP embedding and unsupervised clustering. Differential expression analysis was performed using the FindMarkers function of Seurat with default parameters. Geneset enrichment analysis was performed with the R package clusterProfiler^98^ (v4.8.2) using significantly upregulated genes compared to time-matched vehicle control (abs(avg_log2FC) > 0.5, Benjamini Hochberg FDR < 0.05).

### Transcription factor enrichment analysis

Significantly upregulated genes (avg_log2FC > 0.5, Benjamini Hochberg FDR < 0.05) were computed for paclitaxel, IFNB and IFNG treated samples compared to time-matched vehicle treated cells. ChEA3 enrichment analysis was performed with default settings using R code from the CHEA3 API documentation (https://maayanlab.cloud/chea3/) to perform an online query using either the genes uniquely upregulated in paclitaxel treated cells, or those shared between paclitaxel and either of the interferon responses. The top 15 ranked transcription factors from both the paclitaxel unique and paclitaxel-interferon shared TF enrichment lists were considered when nominating siRNA knockdown targets. Any TF that also had at least 0.25 log2 fold change for paclitaxel at either 24 or 72 h compared to vehicle control was included in the siRNA knockdown panel.

### siRNA knockdown

Cells were plated in 90ul of serum free media per well of a 96 well plate. 24 h later, siRNA knockdown mixture was prepared using a cell-line optimized concentration of Lipofectamine RNAiMAX (cat 13,778,075–075, Invitrogen) and siRNA (Horizon Discovery ON-TARGETplus) following RNAiMAX recommended protocol. The final concentration of siRNA per well was 1 pmol and the final volume of RNAiMAX per well was 75nL for HCC1143, and 37.5nL for HCC1806 or MDA-MB-468 in 100uL of cell containing volume. 24 h after siRNA transfection cells were treated with an addition of 100uL complete media containing either DMSO vehicle control or paclitaxel. The Z’-factor was positive for each cell-line by drug combination, indicating a valid screening assay with sufficient dynamic range between positive and negative controls (Supplemental Data 5)^99^.

### Protein isolation

Protein isolation: siRNA knockdown of HCC1143 cells was performed using the siNonTarget, siELF3, siFOSL1, and siNFE2L2 pools as described above. After 24 h of knockdown, perturbation containing media was added such that media volume doubled and had a final concentration of either 0.1% DMSO (vehicle control) or 1 nM Paclitaxel. After 72 h of perturbation, cells were washed with 4C PBS then lysed by 5 min incubation at 4C with RIPA buffer (R0278, Sigma) supplemented with 1X Halt Protease and Phosphatase Inhibitor Cocktail (1861281, Thermo Scientific). Remaining cells were scraped from the plate and lysate was snap frozen in liquid nitrogen then stored at − 80C overnight. The following day lysate was clarified by centrifugation at ×21,130*g* for 10 min at 4C. The supernatant was collected and the protein concentration was immediately quantified. Remaining protein was stored at − 80C.

### Western blot

Protein quantification was performed using the Western Simple protocol on the Jess capillary western machine using the 12–230 kDa cartridge and following manufacturer instructions (SM-W002, Biotechne). Primary antibodies targeting the protein products of ELF3 (anti-ESE1, NBP3-32320, Novus), FOSL1 (anti-FRA1, #5281S, Cell Signaling Technology), and NFE2L2 (anti-NRF2, #20733S, Cell Signaling Technology) were used at 1:50 dilution. Lysates were loaded at a concentration of 2 mg/mL and volume of 5uL per capillary well, and the Anti-rabbit detection kit (DM-001, Biotechne). was used to quantify primary antibody levels. The Total Protein Detection Module (DM-TP01) was used to normalize input protein across all lanes. Quantification was performed using the included Compass software with default settings (v6.3.0, Biotechne), and the resultant peak areas was plotted using R (v4.3.1).

### HDHB reporter live-cell assays

siRNA knockdown and drug treatment was performed as described above, and then the plate was loaded on an Incucyte S3 (Sartorious) and cells imaged every 15 min for 72 h post drug treatment. At each timepoint 4 fields of view were captured at 20 × magnification in each well using the phase, red and green channels. A cytoplasmic mask was computed from the mean of normalized red/green channel, and a nuclear mask was computed from the red channel using custom trained Cellpose^47^ models. Image quantification was performed in R (v4.3.1) using EBImage (v4.42.0). An additional perinuclear ring mask was computed as the 11 pixel dilation from the nuclear mask, but still bound by the cytoplasmic mask. To determine mClover localization thresholds for cell cycle assignment, 250 cell images were randomly selected and manually assigned to the G1, S/G2 or M cell cycle state based on mClover localization. The mClover intensity ratios were then used to determine thresholds for automated cell cycle phase calling which was applied to the rest of the data set (Supplemental Fig. 5A). Mononuclear cells with a Perinuclear:Nuclear mean intensity ratio greater than 0.8 and Nuclear:Cytoplasmic total intensity less than 0.5 were assigned to the S/G2 phase. Mononuclear and Multinuclear cells with a Nuclear:Cytoplasmic total intensity ratio greater than 0.8 and Perinuclear:Nuclear mean intensity ratio less than 0.8 were assigned to the ‘M’ phase. The remainder of mononuclear cells were assigned ‘G1’, and the remainder of multinucleated cells were assigned ‘Multinucleated’.

### Markov modeling

The 5-frame moving average of cell count per cell cycle phase was down sampled to one value per hour and used to train a Markov model for each unique siRNA (NonTarget, ELF3, FOSL1, NFE2L2, IRF9, PLK1) +/− paclitaxel condition. The transition matrix of the model was constrained such that cells could remain in their current phase, progress through the cell cycle (G1—> S/G2, S/G2—> M, M—> G1 with replication) or transition from M phase to an absorbing (permanent) multinucleated phase. Models were trained for 15 epochs, and the first epoch was seeded with an identity transition matrix. 3000 random transition matrices were generated each epoch, and the 5 with lowest error were used as seeds for the following epoch. The prior best performing matrices were updated with randomly generated matrices at a learning rate of 0.1 for the first epoch, halving every 2 epochs.

The prediction for counts for each future state (S_n+1_) is calculated as the product of the counts at the prior state (S_n_) by the transition matrix (P) and the replication matrix (RM).

$$RM= \begin{array}{ccccc} & G1& S/G2& M& Multi.\\ G1& 1& 1& 0& 0\\ S/G2& 0& 1& 1& 0\\ M& 2& 0& 1& 1\\ Multi.& 0& 0& 0& 1\end{array}$$

$$P = \begin{array}{ccccc} & G1& S/G2& M& Multi.\\ G1& ?& ?& 0& 0\\ S/G2& 0& ?& ?& 0\\ M& ?& 0& ?& ?\\ Multi.& 0& 0& 0& ?\end{array}$$

$${S}_{n+1}={S}_{n}*P*RM$$

The error of the Markov predicted cell counts (c_exp_) compared to observed counts (c_obs_) was computed as the arithmetic mean of the Root Mean Squared Relative Error (RMSRE) of each cell cycle phase across all predicted timepoints. The noise floor of RMSRE was estimated with a second-order loess fit with span of 0.75 (loess function from R package ‘stats’, v4.3.1).

$$RMSRE= \sqrt{\frac{1}{n}*\sum \frac{{({c}_{exp}-{c}_{obs})}^{2}}{{{c}_{obs}}^{2}}}$$

The mitotic success rate (MSR) of each condition was computed as the ratio of M-to-G1 transition (P_M,G1_) to the sum of the transition rates for M-to-G1 (P_M,G1_) and M-to-multinucleated (P_M,Multi_):

$$MSR=\frac{{P}_{M,G1}}{{P}_{M,G1}+{P}_{M,Multi}}$$

The expected duration of G1, S/G2 and M cell cycle phases was calculated from the homotypic transition rates as^100^:

$$For (i==j): {ExpectedDuration}_{i,j}= \frac{1}{1-{P}_{i,j}}$$

### METABRIC survival and microarray analysis

The METABRIC^50^ microarray and patient metadata was accessed through cbioportal^101–103^ and analyzed using R (v4.3.2) and the ‘survival’ package (v3.5.7). The z-scored microarray expression data was used to categorize patients into ‘high’ (highest expressing quartile), ‘mid’ (first to third expressing quartile) or ‘low’ (lowest expressing quartile) based on expression of ELF3. For survival analysis, patients were filtered to those with microarray data and then Kaplan–meier survival curves were generated with the ‘ggsurvfit’ package (v1.0.0). Cox proportional hazard statistics were calculated with the ‘coxph’ function of the ‘survival’ package (v3.5.7). Differential expression was calculated from the log normalized microarray data using the ‘wilcoxauc’ function from the ‘presto’ package (v1.0.0). Significantly differentially expressed genes (abs(logFC) > 0.5 and adjusted *p* < 0.05) where used to compute MSigDB hallmark GSEA using the ‘clusterprofiler’ (v4.10.1) and ‘msigdbr’ (v7.5.1) packages.

### Full reagent list

**Table:** 

Reagent	Shorthand	Type	Source	Identifier
HCC1143	–	Cell line	ATCC	CRL-2321
HCC1806	–	Cell line	ATCC	CRL-2335
MDA-MB-468	–	Cell line	ATCC	HTB-132
RPMI 1640	RPMI	Reagent	Life Technologies	11875119
DMEM	DMEM	Reagent	Life Technologies	11965-092
Fetal Bovine Serum	FBS	Reagent	Gibco	16000-044
Dimethyl Sulfoxide	DMSO	Reagent	Millipore Sigma	D8418-250ML
Phosphate Buffered Saline	PBS	Reagent	Gibco	14190235
Paclitaxel	PTX	Reagent	LC Labs	P-9600
Interferon-Beta	IFNB	Reagent	PBL Assay Science	11410-2
Interferon-Gamma	IFNG	Reagent	R&D Systems	385-IR-100
Human Oncostatin M	OSM	reagent	Cell Signaling Technology	5367SC
Recombinant Human Lymphotoxin alpha1/beta2 protein	LT	Reagent	Biotechne	8884-LY-025
Recombinant Human TGFB-Beta 1	TGFB	Reagent	Biotechne	7754BH005
BMS-906024	NOTCHi	Reagent	Millipore Sigma	BM0018-5MG
16% Formaldehyde (w/v)	–	Reagent	ThermoFisher Scientific	28,908
Triton X-100	–	Reagent	Millipore Sigma	X100-100ML
Normal Goat Serum Blocking Solution	–	Reagent	MP Biomedicals	#0219135680
Lipofectamine RNAiMAX transfection Reagent	–	Reagent	ThermoFisher Scientific	13778075
Bovine Serum Albumin	BSA	Reagent	Millipore Sigma	A7906-100G
RIPA buffer	–	Reagent	Sigma	P0278
100X Halt Protease and Phosphatase Inhibitor Cocktail	–	Reagent	ThermoFisher Scientific	1861281
Click-iT Plus EdU Cell Proliferation kit (AF647)	–	Reagent	Invitrogen	C10640
anti-CDKN2A/p16INK4A + CDKN2B/p15INK4B-AF644	p16	Antibody	Abcam	ab199756
anti-cPARP-AF647	cPARP	antibody	Cell Signaling Technology	6987S
anti-TUBB3-AF647	TUBB3	Antibody	Abcam	ab190575
HCS CellMask Green	CellMask Green	Stain	Invitrogen	H32713
HCS CellMask Orange	CellMask Orange	Stain	Invitrogen	H32714
DAPI	DAPI	Stain	Cell Signaling Technology	4083S
ELF3/ESE-1 Antibody	–	Antibody	Novus	NBP3-32320
FRA1 (D80BP) Rabbit mAb	–	Antibody	Cell Signaling Technology	5281
NRF2 (E5F1A) Rabbit mAb	–	Antibody	Cell Signaling Technology	20733
12–230 kDa separation module	–	Reagent	Biotechne	SM-W002
Total Protein Detection Module	–	Reagent	Biotechne	DM-TP01
Anti-Rabbit Detection Module	–	Reagent	Biotechne	DM-001
siATF3 Smartpool	siATF3	siRNA	Hoizon Discovery	L-008663-00
siDDIT3 Smartpool	siDDIT3	siRNA	Hoizon Discovery	L-004819-00
siELF3 Smartpool	siELF3	siRNA	Hoizon Discovery	L-016080-00
siFOSL1 Smartpool	siFOSL1	siRNA	Hoizon Discovery	L-004341-00
siIRF7 Smartpool	siIRF7	siRNA	Hoizon Discovery	L-011810-00
siIRF9 Smartpool	siIRF9	siRNA	Hoizon Discovery	L-020858-00
siJUNB Smartpool	siJUNB	siRNA	Hoizon Discovery	L-003269-00
siJUN Smartpool	siJUN	siRNA	Hoizon Discovery	L-003268-00
siKIF11 Smartpool	siKIF11	siRNA	Hoizon Discovery	L-003317-00
siKLF6 Smartpool	siKLF6	siRNA	Hoizon Discovery	L-021441-00
siMAFF Smartpool	siMAFF	siRNA	Hoizon Discovery	L-003903-00
siNFE2L2 Smartpool	siNFE2L2	siRNA	Hoizon Discovery	L-003755-00
siPLK1 Smartpool	siPLK1	siRNA	Hoizon Discovery	L-003290-00
siPLSCR1 Smartpool	siPLSCR1	siRNA	Hoizon Discovery	L-003729-00
siSP100 Smartpool	siSP100	siRNA	Hoizon Discovery	L-015307-00
ON-TARGETplus Non-targeting Control	siNonTarget	siRNA	Hoizon Discovery	D-001810-10
ON-TARGETplus GAPD Control	siGAPD	siRNA	Hoizon Discovery	D-001830-10

## Supplementary Information

Supplementary Information 1.

Supplementary Information 2.

Supplementary Information 3.

Supplementary Information 4.

Supplementary Information 5.

Supplementary Information 6.

## Data Availability

Single Cell RNA-seq data is available on the Gene Expression Omnibus with study ID: GSE266934 (https://www.ncbi.nlm.nih.gov/geo/query/acc.cgi?acc=GSE266934). The images and processed data from immunofluorescent stained HCC1143 are available on Zenodo: (10.5281/zenodo.11237850). The images and processed data from the siRNA panel, and the processed data from live-cell imaging study are available on Zenodo (10.5281/zenodo.11238552). The images and processed data from EdU incorporation assays are available on Zenodo (10.5281/zenodo.14226249). The images from the live-cell experiments are available upon request.

## Code availability

All code related to scRNA-seq data processing and figure generation are available on Github (https://github.com/HeiserLab/PTX_manuscript). Code and segmentation models related to image processing and feature extraction are included in the respective Zenodo repository for each experiment.

## References

[1] Bauer, K. R. et al. Descriptive analysis of estrogen receptor (ER)-negative, progesterone receptor (PR)-negative, and HER2-negative invasive breast cancer, the so-called triple-negative phenotype. *Cancer* **109**(9), 1721–1728 (2007). 17387718 10.1002/cncr.22618

[2] Early Breast Cancer Trialists’ Collaborative Group. Comparisons between different polychemotherapy regimens for early breast cancer: Meta-analyses of long-term outcome among 100 000 women in 123 randomised trials. *The Lancet* **379**(9814), 432–444 (2012). 10.1016/S0140-6736(11)61625-5 PMC3273723 22152853

[3] Mustacchi, G. & De Laurentiis, M. The role of taxanes in triple-negative breast cancer: Literature review. *Drug Des. Dev. Ther.* **9**, 4303 (2015). 10.2147/DDDT.S86105 PMC4532347 26273192

[4] Abu Samaan, T. M. et al. Paclitaxel’s mechanistic and clinical effects on breast cancer. *Biomolecules* **9**(12), 789 (2019). 31783552 10.3390/biom9120789 PMC6995578

[5] Hu, Y. et al. Paclitaxel induces micronucleation and activates pro-inflammatory cGAS-STING signaling in triple-negative breast cancer. *Mol. Cancer Ther.* **20**(12), 2553–2567 (2021). 34583980 10.1158/1535-7163.MCT-21-0195 PMC8643310

[6] Smith, E. R. & Xu, X. X. Breaking malignant nuclei as a non-mitotic mechanism of taxol/paclitaxel. *J. Cancer Biol.* **2**(4), 86–93 (2021). 35048083 10.46439/cancerbiology.2.031 PMC8765745

[7] Liedtke, C. et al. Response to neoadjuvant therapy and long-term survival in patients with triple-negative breast cancer. *J. Clin. Oncol.* **41**(10), 1809–1815 (2023). 10.1200/JCO.2007.14.4147 18250347

[8] Foulkes, W. D., Smith, I. E. & Reis-Filho, J. S. Triple-negative breast cancer. *N. Engl. J. Med.* **363**(20), 1938–1948 (2010). 21067385 10.1056/NEJMra1001389

[9] Schmid, P. et al. Atezolizumab and nab-paclitaxel in advanced triple-negative breast cancer. *N. Engl. J. Med.* **379**(22), 2108–2121 (2018). 30345906 10.1056/NEJMoa1809615

[10] Labrie, M. et al. Therapy resistance: Opportunities created by adaptive responses to targeted therapies in cancer. *Nat. Rev. Cancer* **22**(6), 323–339 (2022). 35264777 10.1038/s41568-022-00454-5 PMC9149051

[11] Burstein, M. D. et al. Comprehensive genomic analysis identifies novel subtypes and targets of triple-negative breast cancer. *Clin. Cancer Res.* **21**(7), 1688–1698 (2015). 25208879 10.1158/1078-0432.CCR-14-0432 PMC4362882

[12] Spranger, S. & Gajewski, T. F. Impact of oncogenic pathways on evasion of antitumour immune responses. *Nat. Rev. Cancer* **18**(3), 139–147 (2018). 29326431 10.1038/nrc.2017.117 PMC6685071

[13] Baillie, K. E. & Stirling, P. C. Beyond kinases: Targeting replication stress proteins in cancer therapy. *Trends Cancer* **7**(5), 430–446 (2021). 33203609 10.1016/j.trecan.2020.10.010

[14] Nguyen, C. D. K. & Yi, C. YAP/TAZ signaling and resistance to cancer therapy. *Trends Cancer* **5**(5), 283–296 (2019). 31174841 10.1016/j.trecan.2019.02.010 PMC6557283

[15] Lawson, D. A. et al. Tumour heterogeneity and metastasis at single-cell resolution. *Nat. Cell Biol.* **20**(12), 1349–1360 (2018). 30482943 10.1038/s41556-018-0236-7 PMC6477686

[16] Aibar, S. et al. SCENIC: Single-cell regulatory network inference and clustering. *Nat. Methods* **14**(11), 1083–1086 (2017). 28991892 10.1038/nmeth.4463 PMC5937676

[17] Obradovic, A. et al. Single-cell protein activity analysis identifies recurrence-associated renal tumor macrophages. *Cell* **184**(11), 2988-3005 e16 (2021). 34019793 10.1016/j.cell.2021.04.038 PMC8479759

[18] Hafner, M. et al. Growth rate inhibition metrics correct for confounders in measuring sensitivity to cancer drugs. *Nat. Methods* **13**(6), 521–527 (2016). 27135972 10.1038/nmeth.3853 PMC4887336

[19] Weaver, B. A. How Taxol/paclitaxel kills cancer cells. *Mol. Biol. Cell* **25**(18), 2677–2681 (2014). 25213191 10.1091/mbc.E14-04-0916 PMC4161504

[20] Lee, K. M. et al. Class III β-tubulin, a marker of resistance to paclitaxel, is overexpressed in pancreatic ductal adenocarcinoma and intraepithelial neoplasia. *Histopathology* **51**(4), 539–546 (2007). 17714470 10.1111/j.1365-2559.2007.02792.x

[21] Stengel, C. et al. Class III beta-tubulin expression and in vitro resistance to microtubule targeting agents. *Br. J. Cancer* **102**(2), 316–324 (2010). 20029418 10.1038/sj.bjc.6605489 PMC2816659

[22] Sawant, K. V. et al. Chemokine CXCL1 mediated neutrophil recruitment: Role of glycosaminoglycan interactions. *Sci. Rep.* **6**, 33123 (2016). 27625115 10.1038/srep33123 PMC5021969

[23] Cambier, S., Gouwy, M. & Proost, P. The chemokines CXCL8 and CXCL12: Molecular and functional properties, role in disease and efforts towards pharmacological intervention. *Cell Mol. Immunol.* **20**(3), 217–251 (2023). 36725964 10.1038/s41423-023-00974-6 PMC9890491

[24] Xiong, X. et al. CXCL8 in tumor biology and its implications for clinical translation. *Front. Mol. Biosci.* **9**, 723846 (2022). 35372515 10.3389/fmolb.2022.723846 PMC8965068

[25] Wang, N. et al. CXCL1 derived from tumor-associated macrophages promotes breast cancer metastasis via activating NF-kappaB/SOX4 signaling. *Cell Death Dis.* **9**(9), 880 (2018). 30158589 10.1038/s41419-018-0876-3 PMC6115425

[26] Gross, S. M. et al. A multi-omic analysis of MCF10A cells provides a resource for integrative assessment of ligand-mediated molecular and phenotypic responses. *Commun. Biol.* **5**(1), 1066 (2022). 36207580 10.1038/s42003-022-03975-9 PMC9546880

[27] Krishna, B. M. et al. Notch signaling in breast cancer: From pathway analysis to therapy. *Cancer Lett.* **461**, 123–131 (2019). 31326555 10.1016/j.canlet.2019.07.012 PMC9003668

[28] Arico, E. et al. Type I interferons and cancer: An evolving story demanding novel clinical applications. *Cancers (Basel)* **11**(12), 1943 (2019). 31817234 10.3390/cancers11121943 PMC6966569

[29] Jorgovanovic, D. et al. Roles of IFN-gamma in tumor progression and regression: A review. *Biomark. Res.* **8**, 49 (2020). 33005420 10.1186/s40364-020-00228-x PMC7526126

[30] Chiu, Y. H., Macmillan, J. B. & Chen, Z. J. RNA polymerase III detects cytosolic DNA and induces type I interferons through the RIG-I pathway. *Cell* **138**(3), 576–591 (2009). 19631370 10.1016/j.cell.2009.06.015 PMC2747301

[31] Yu, L. & Liu, P. Cytosolic DNA sensing by cGAS: Regulation, function, and human diseases. *Signal Transduct. Target Ther.* **6**(1), 170 (2021). 33927185 10.1038/s41392-021-00554-y PMC8085147

[32] Keenan, A. B. et al. ChEA3: Transcription factor enrichment analysis by orthogonal omics integration. *Nucleic Acids Res.* **47**(W1), W212–W224 (2019). 31114921 10.1093/nar/gkz446 PMC6602523

[33] Bahrami, S. & Drablos, F. Gene regulation in the immediate-early response process. *Adv. Biol. Regul.* **62**, 37–49 (2016). 27220739 10.1016/j.jbior.2016.05.001

[34] Jefferies, C. A. Regulating IRFs in IFN driven disease. *Front. Immunol.* **10**, 325 (2019). 30984161 10.3389/fimmu.2019.00325 PMC6449421

[35] Guler, R. et al. Batf2 differentially regulates tissue immunopathology in Type 1 and Type 2 diseases. *Mucosal Immunol.* **12**(2), 390–402 (2019). 30542107 10.1038/s41385-018-0108-2 PMC7051910

[36] Liu, J. et al. Batf3(+) DCs and type I IFN are critical for the efficacy of neoadjuvant cancer immunotherapy. *Oncoimmunology* **8**(2), e1546068 (2019). 30713806 10.1080/2162402X.2018.1546068 PMC6343771

[37] Rehwinkel, J. & Gack, M. U. RIG-I-like receptors: Their regulation and roles in RNA sensing. *Nat. Rev. Immunol.* **20**(9), 537–551 (2020). 32203325 10.1038/s41577-020-0288-3 PMC7094958

[38] de Garces Los Fayos Alonso, I. et al. The role of activator protein-1 (AP-1) family members in CD30-positive lymphomas. *Cancers (Basel)* **10**(4), 93 (2018). 29597249 10.3390/cancers10040093 PMC5923348

[39] Otero, M. et al. E74-like factor 3 (ELF3) impacts on matrix metalloproteinase 13 (MMP13) transcriptional control in articular chondrocytes under proinflammatory stress. *J. Biol. Chem.* **287**(5), 3559–3572 (2012). 22158614 10.1074/jbc.M111.265744 PMC3271009

[40] Kim, T. H., Kern, C. & Zhou, H. Knockout of IRF7 highlights its modulator function of host response against avian influenza virus and the involvement of MAPK and TOR signaling pathways in chicken. *Genes (Basel)* **11**(4), 385 (2020). 32252379 10.3390/genes11040385 PMC7230310

[41] Li, T. et al. DDIT3 and KAT2A proteins regulate TNFRSF10A and TNFRSF10B expression in endoplasmic reticulum stress-mediated apoptosis in human lung cancer cells. *J. Biol. Chem.* **290**(17), 11108–11118 (2015). 25770212 10.1074/jbc.M115.645333 PMC4409269

[42] Belanova, A. A. et al. Effects of JUN and NFE2L2 knockdown on oxidative status and NFE2L2/AP-1 targets expression in HeLa cells in basal conditions and upon sub-lethal hydrogen peroxide treatment. *Mol. Biol. Rep.* **46**(1), 27–39 (2019). 30515697 10.1007/s11033-018-4412-4

[43] Mirzaei, H. et al. The AP-1 pathway; A key regulator of cellular transformation modulated by oncogenic viruses. *Rev. Med. Virol.* **30**(1), e2088 (2020). 31788897 10.1002/rmv.2088

[44] Gross, S. M. et al. Analysis and modeling of cancer drug responses using cell cycle phase-specific rate effects. *Nat. Commun.* **14**(1), 3450 (2023). 37301933 10.1038/s41467-023-39122-z PMC10257663

[45] Spencer, S. L. et al. The proliferation-quiescence decision is controlled by a bifurcation in CDK2 activity at mitotic exit. *Cell* **155**(2), 369–383 (2013). 24075009 10.1016/j.cell.2013.08.062 PMC4001917

[46] Luker, K. E. et al. Overexpression of IRF9 confers resistance to antimicrotubule agents in breast cancer cells1. *Cancer Res.* **61**(17), 6540–6547 (2001). 11522652

[47] Stringer, C. et al. Cellpose: A generalist algorithm for cellular segmentation. *Nat. Methods* **18**(1), 100–106 (2021). 33318659 10.1038/s41592-020-01018-x

[48] Mohammadi, F. et al. A lineage tree-based hidden Markov model quantifies cellular heterogeneity and plasticity. *Commun. Biol.* **5**(1), 1258 (2022). 36396800 10.1038/s42003-022-04208-9 PMC9671968

[49] Palmer, A. C. & Sorger, P. K. Combination cancer therapy can confer benefit via patient-to-patient variability without drug additivity or synergy. *Cell* **171**(7), 1678-1691 e13 (2017). 29245013 10.1016/j.cell.2017.11.009 PMC5741091

[50] Curtis, C. et al. The genomic and transcriptomic architecture of 2,000 breast tumours reveals novel subgroups. *Nature* **486**(7403), 346–352 (2012). 22522925 10.1038/nature10983 PMC3440846

[51] Symmans, W. F. et al. Long-term prognostic risk after neoadjuvant chemotherapy associated with residual cancer burden and breast cancer subtype. *J. Clin. Oncol.* **35**(10), 1049–1060 (2017). 28135148 10.1200/JCO.2015.63.1010 PMC5455352

[52] Foo, J. & Michor, F. Evolution of acquired resistance to anti-cancer therapy. *J. Theor. Biol.* **355**, 10–20 (2014). 24681298 10.1016/j.jtbi.2014.02.025 PMC4058397

[53] Nussinov, R., Tsai, C. J. & Jang, H. Anticancer drug resistance: An update and perspective. *Drug Resist. Updat.* **59**, 100796 (2021). 34953682 10.1016/j.drup.2021.100796 PMC8810687

[54] Lukow, D. A. et al. Chromosomal instability accelerates the evolution of resistance to anti-cancer therapies. *Dev. Cell* **56**(17), 2427-2439 e4 (2021). 34352222 10.1016/j.devcel.2021.07.009 PMC8933054

[55] Hashemi, M. et al. EMT mechanism in breast cancer metastasis and drug resistance: Revisiting molecular interactions and biological functions. *Biomed. Pharmacother.* **155**, 113774 (2022). 36271556 10.1016/j.biopha.2022.113774

[56] Heiser, L. M. et al. Subtype and pathway specific responses to anticancer compounds in breast cancer. *Proc. Natl. Acad. Sci. U S A* **109**(8), 2724–2729 (2012). 22003129 10.1073/pnas.1018854108 PMC3286973

[57] Barretina, J. et al. The cancer cell line Encyclopedia enables predictive modelling of anticancer drug sensitivity. *Nature* **483**(7391), 603–607 (2012). 22460905 10.1038/nature11003 PMC3320027

[58] Yang, W. et al. Genomics of drug sensitivity in cancer (GDSC): A resource for therapeutic biomarker discovery in cancer cells. *Nucleic Acids Res.* **41**(Database issue), D955–D961 (2013). 23180760 10.1093/nar/gks1111 PMC3531057

[59] Chan, Y. T. et al. CRISPR-Cas9 library screening approach for anti-cancer drug discovery: Overview and perspectives. *Theranostics* **12**(7), 3329–3344 (2022). 35547744 10.7150/thno.71144 PMC9065202

[60] Guillon, J. et al. Chemotherapy-induced senescence, an adaptive mechanism driving resistance and tumor heterogeneity. *Cell Cycle* **18**(19), 2385–2397 (2019). 31397193 10.1080/15384101.2019.1652047 PMC6738909

[61] Kim, C. J. et al. Nuclear morphology predicts cell survival to cisplatin chemotherapy. *Neoplasia* **42**, 100906 (2023). 37172462 10.1016/j.neo.2023.100906 PMC10314150

[62] Pargett, M. et al. Single-cell imaging of ERK signaling using fluorescent biosensors. *Methods Mol. Biol.* **1636**, 35–59 (2017). 28730471 10.1007/978-1-4939-7154-1_3 PMC8005261

[63] Netterfield, T. S. et al. Biphasic JNK-Erk signaling separates the induction and maintenance of cell senescence after DNA damage induced by topoisomerase II inhibition. *Cell Syst.* **14**(7), 582-604 e10 (2023). 37473730 10.1016/j.cels.2023.06.005 PMC10627503

[64] Ender, P. et al. Spatiotemporal control of ERK pulse frequency coordinates fate decisions during mammary acinar morphogenesis. *Dev. Cell* **57**(18), 2153-2167 e6 (2022). 36113484 10.1016/j.devcel.2022.08.008

[65] Regot, S. et al. High-sensitivity measurements of multiple kinase activities in live single cells. *Cell* **157**(7), 1724–1734 (2014). 24949979 10.1016/j.cell.2014.04.039 PMC4097317

[66] Grant, G. D. et al. Accurate delineation of cell cycle phase transitions in living cells with PIP-FUCCI. *Cell Cycle* **17**(21–22), 2496–2516 (2018). 30421640 10.1080/15384101.2018.1547001 PMC6342071

[67] Wang, Y. et al. Real-time imaging of senescence in tumors with DNA damage. *Sci. Rep.* **9**(1), 2102 (2019). 30765819 10.1038/s41598-019-38511-z PMC6375927

[68] Balderstone, L. A. et al. Development of a fluorescence-based cellular apoptosis reporter. *Methods Appl. Fluoresc.* **7**(1), 015001 (2018). 30353887 10.1088/2050-6120/aae6f8 PMC6372133

[69] Nam, A. S., Chaligne, R. & Landau, D. A. Integrating genetic and non-genetic determinants of cancer evolution by single-cell multi-omics. *Nat. Rev. Genet.* **22**(1), 3–18 (2021). 32807900 10.1038/s41576-020-0265-5 PMC8450921

[70] Schiff, P. B., Fant, J. & Horwitz, S. B. Promotion of microtubule assembly in vitro by taxol. *Nature* **277**(5698), 665–667 (1979). 423966 10.1038/277665a0

[71] de Visser, K. E. & Joyce, J. A. The evolving tumor microenvironment: From cancer initiation to metastatic outgrowth. *Cancer Cell* **41**(3), 374–403 (2023). 36917948 10.1016/j.ccell.2023.02.016

[72] Khalaf, K. et al. Aspects of the tumor microenvironment involved in immune resistance and drug resistance. *Front. Immunol.* **12**, 656364 (2021). 34122412 10.3389/fimmu.2021.656364 PMC8190405

[73] Deepak, K. G. K. et al. Tumor microenvironment: Challenges and opportunities in targeting metastasis of triple negative breast cancer. *Pharmacol. Res.* **153**, 104683 (2020). 32050092 10.1016/j.phrs.2020.104683

[74] Ma, L. et al. Tumor cell biodiversity drives microenvironmental reprogramming in liver cancer. *Cancer Cell* **36**(4), 418-430 e6 (2019). 31588021 10.1016/j.ccell.2019.08.007 PMC6801104

[75] Pitt, J. M. et al. Targeting the tumor microenvironment: Removing obstruction to anticancer immune responses and immunotherapy. *Ann. Oncol.* **27**(8), 1482–1492 (2016). 27069014 10.1093/annonc/mdw168

[76] Xiao, Y. & Yu, D. Tumor microenvironment as a therapeutic target in cancer. *Pharmacol. Ther.* **221**, 107753 (2021). 33259885 10.1016/j.pharmthera.2020.107753 PMC8084948

[77] Hu, J. et al. Regulation of tumor immune suppression and cancer cell survival by CXCL1/2 elevation in glioblastoma multiforme. *Sci. Adv.* **7**(5), eabc2511 (2021). 33571109 10.1126/sciadv.abc2511 PMC7840139

[78] Yang, M. et al. Tumour-associated neutrophils orchestrate intratumoural IL-8-driven immune evasion through Jagged2 activation in ovarian cancer. *Br. J. Cancer* **123**(9), 1404–1416 (2020). 32778818 10.1038/s41416-020-1026-0 PMC7591527

[79] Heidemann, J. et al. Angiogenic effects of interleukin 8 (CXCL8) in human intestinal microvascular endothelial cells are mediated by CXCR2. *J. Biol. Chem.* **278**(10), 8508–8515 (2003). 12496258 10.1074/jbc.M208231200

[80] Choi, H. S. et al. Disruption of the NF-kappaB/IL-8 signaling axis by sulconazole inhibits human breast cancer stem cell formation. *Cells* **8**(9), 1007 (2019). 31480284 10.3390/cells8091007 PMC6770215

[81] Rhodes, D. R. et al. Mining for regulatory programs in the cancer transcriptome. *Nat. Genet.* **37**(6), 579–583 (2005). 15920519 10.1038/ng1578

[82] Margolin, A. A. et al. ARACNE: an algorithm for the reconstruction of gene regulatory networks in a mammalian cellular context. *BMC Bioinform.* **7**(Suppl 1), S7 (2006). 10.1186/1471-2105-7-S1-S7 PMC1810318 16723010

[83] Wang, K. et al. Genome-wide identification of post-translational modulators of transcription factor activity in human B cells. *Nat. Biotechnol.* **27**(9), 829–839 (2009). 19741643 10.1038/nbt.1563 PMC2753889

[84] Subbalakshmi, A. R. et al. The ELF3 transcription factor is associated with an epithelial phenotype and represses epithelial-mesenchymal transition. *J. Biol. Eng.* **17**(1), 17 (2023). 36864480 10.1186/s13036-023-00333-z PMC9983220

[85] Enfield, K. S. S. et al. Epithelial tumor suppressor ELF3 is a lineage-specific amplified oncogene in lung adenocarcinoma. *Nat. Commun.* **10**(1), 5438 (2019). 31780666 10.1038/s41467-019-13295-y PMC6882813

[86] Horie, M. et al. An integrative epigenomic approach identifies ELF3 as an oncogenic regulator in ASCL1-positive neuroendocrine carcinoma. *Cancer Sci.* **114**(6), 2596–2608 (2023). 36840413 10.1111/cas.15764 PMC10236617

[87] Archer, L. K. et al. ETS transcription factor ELF3 (ESE-1) is a cell cycle regulator in benign and malignant prostate. *FEBS Open Bio* **12**(7), 1365–1387 (2022). 35472129 10.1002/2211-5463.13417 PMC9249341

[88] Fina, E. et al. Gene signatures of circulating breast cancer cell models are a source of novel molecular determinants of metastasis and improve circulating tumor cell detection in patients. *J. Exp. Clin. Cancer Res.* **41**(1), 78 (2022). 35216615 10.1186/s13046-022-02259-8 PMC8876758

[89] Mermel, C. H. et al. GISTIC2.0 facilitates sensitive and confident localization of the targets of focal somatic copy-number alteration in human cancers. *Genome Biol.* **12**(4), R41 (2011). 21527027 10.1186/gb-2011-12-4-r41 PMC3218867

[90] Mesquita, B. et al. Frequent copy number gains at 1q21 and 1q32 are associated with overexpression of the ETS transcription factors ETV3 and ELF3 in breast cancer irrespective of molecular subtypes. *Breast Cancer Res. Treat.* **138**(1), 37–45 (2013). 23329352 10.1007/s10549-013-2408-2

[91] Riemenschneider, M. J., Knobbe, C. B. & Reifenberger, G. Refined mapping of 1q32 amplicons in malignant gliomas confirms MDM4 as the main amplification target. *Int. J. Cancer* **104**(6), 752–757 (2003). 12640683 10.1002/ijc.11023

[92] Veerakumarasivam, A. et al. High-resolution array-based comparative genomic hybridization of bladder cancers identifies mouse double minute 4 (MDM4) as an amplification target exclusive of MDM2 and TP53. *Clin. Cancer Res.* **14**(9), 2527–2534 (2008). 18451213 10.1158/1078-0432.CCR-07-4129

[93] Francois, M., Donovan, P. & Fontaine, F. Modulating transcription factor activity: Interfering with protein-protein interaction networks. *Semin. Cell Dev. Biol.* **99**, 12–19 (2020). 30172762 10.1016/j.semcdb.2018.07.019

[94] Ngamcherdtrakul, W. & Yantasee, W. siRNA therapeutics for breast cancer: Recent efforts in targeting metastasis, drug resistance, and immune evasion. *Transl. Res.* **214**, 105–120 (2019). 31487500 10.1016/j.trsl.2019.08.005 PMC6848785

[95] Wolpaw, A. J. et al. Drugging the “Undruggable” MYCN oncogenic transcription factor: Overcoming previous obstacles to impact childhood cancers. *Cancer Res.* **81**(7), 1627–1632 (2021). 33509943 10.1158/0008-5472.CAN-20-3108 PMC8392692

[96] Butler, A. et al. Integrating single-cell transcriptomic data across different conditions, technologies, and species. *Nat. Biotechnol.* **36**(5), 411–420 (2018). 29608179 10.1038/nbt.4096 PMC6700744

[97] Satija, R. et al. Spatial reconstruction of single-cell gene expression data. *Nat. Biotechnol.* **33**(5), 495–502 (2015). 25867923 10.1038/nbt.3192 PMC4430369

[98] Yu, G. et al. clusterProfiler: An R package for comparing biological themes among gene clusters. *OMICS J. Integr. Biol.* **16**(5), 284–287 (2012). 10.1089/omi.2011.0118 PMC3339379 22455463

[99] Zhang, J. H., Chung, T. D. & Oldenburg, K. R. A simple statistical parameter for use in evaluation and validation of high throughput screening assays. *J. Biomol. Screen.* **4**(2), 67–73 (1999). 10838414 10.1177/108705719900400206

[100] Dudel, C. & Myrskylä, M. Estimating the number and length of episodes in disability using a Markov chain approach. *Popul. Health Metrics* **18**(1), 1 (2020). 10.1186/s12963-020-00217-0 PMC7389377 32727599

[101] Cerami, E. et al. The cBio cancer genomics portal: An open platform for exploring multidimensional cancer genomics data. *Cancer Discov.* **2**(5), 401–404 (2012). 22588877 10.1158/2159-8290.CD-12-0095 PMC3956037

[102] de Bruijn, I. et al. Analysis and visualization of longitudinal genomic and clinical data from the AACR project GENIE biopharma collaborative in cBioPortal. *Cancer Res.* **83**(23), 3861–3867 (2023). 37668528 10.1158/0008-5472.CAN-23-0816 PMC10690089

[103] Gao, J. et al. Integrative analysis of complex cancer genomics and clinical profiles using the cBioPortal. *Sci. Signal* **6**(269), pl1 (2013). 23550210 10.1126/scisignal.2004088 PMC4160307

