# Supplementary material for: TNBC response to paclitaxel phenocopies interferon response which reveals cell cycle-associated resistance mechanisms
Source: Sci Rep. 2025 Feb 4;15:4294. doi: 10.1038/s41598-024-82218-9 (PMC11794704; doi:10.1038/s41598-024-82218-9)

Supplementary information

TNBC response to paclitaxel phenocopies interferon response which reveals cell cycle-associated resistance mechanisms

Nicholas L Calistri[1], Tiera A. Liby[1], Zhi Hu[1], Hongmei Zhang[1], Mark Dane[1], Sean M. Gross[1], Laura M. Heiser[1,2,#]

[1] Biomedical Engineering Department, Oregon Health & Science University, Portland Oregon

[2] Knight Cancer Institute, Oregon Health & Science University, Portland Oregon

[#] Corresponding author: heiserl@ohsu.edu

Supplementary files

Supplementary Information 1 (This file): contains supplemental figures referenced within the manuscript

Supplemental Information 2: contains differential gene expression results for all scRNA-seq perturbation studies compared to time-matched vehicle control.

Supplemental Information 3: contains ontology enrichment results for all scRNA-seq experiments.

Supplemental Information 4: contains differential gene expression results for between ELF3-high and ELF3-low samples from the Metabric cohort.

Supplemental Information 5: contains MsigDB GSEA results for ELF3-high and ELF3-low samples from the Metabric cohort.

Supplemental Information 6: contains z-factor results for each siRNA experiments.

Supplementary Figure 1: S1A) Scatter plot showing the thresholds for classifying each cell line as 2N/EdU- (bottom left), EdU+ (top) and 4N/EdU- (bottom right). Each plot shows a random sampling of 300 DMSO treated cells. **S1B)** 2D-Density plot showing the distribution of HCC1143 cells for all paclitaxel concentration. X axis shows mean intensity for p15/p16, y axis shows mean Intensity for TUBB3 in arbitrary units (A.U.). Mean intensities were calculated for the complete cellular mask (includes both cytoplasmic and nuclear regions) R^2 squared shown for Pearson correlation (p-value < 2.2e-16). **S1C)** Breakout plots showing the same data as S1B, split for each paclitaxel concentration. Control (DMSO) shown for every inset plot in gray, and paclitaxel in black for the nM dose listed above. **S1D)** Cell cycle state for HCC1143 cells comparing mononucleated vs multinucleated cells.

Supplementary Figure 2: S2A) Violin plots of scRNA-seq QC metrics for the four conditions. Horizontal lines indicate the first, second and third quartiles. **S2B)** Breakout plots showing the same UMAP as Figure 2A split by condition and color coded by cell cycle phase. **S2C,D)** Euler plot showing the overlap in significantly upregulated genes (SF2C) and enriched Reactome pathways (SF2D) between paclitaxel at 24 hours (PTX24) and 72 hours (PTX72) compared to time matched control. **S2E)** Barplots showing mean expression of chemokines CXCL1 and CXCL8 which were significantly upregulated in both paclitaxel conditions compared to time matched control.

Supplementary Figure 3: S3A) Heatmap showing expression for interferon ligands (gray annotation) and receptors (black annotation) for each of the paclitaxel scRNA-seq conditions. **S3B)** Violin plots of scRNA-seq QC metrics for the ligand perturbation conditions. Horizontal lines indicate the first, second and third quartiles.

S3C) The same ligand perturbation scRNA-seq UMAP as Figure 3A, but color coded by cell cycle phase assignment. **S3D)** Bar plot indicating the proportion of cells assigned to each cell cycle phase for each condition. **S3E)** Fraction of multinucleated cells for HCC1143 cells treated with 20ng/mL IFNB, 20ng/mL IFNG or 0.1% PBS for 72 hours then fixed and stained with DAPI and HCS cellmask. **S3F)** Heatmap showing the pearson correlation in single cell gene expression for implicated transcription factors. Data subset to include just paclitaxel treated cells from both time points.

Supplementary Figure 4: S4A) Dotplot showing the expression of implicated TFs for the PBS, IFNB, and IFNG treatment conditions. **S4B)** siRNA knockdown capillary western results using HCC1806 cell line. Spectra images from ProteinSimple/WesternSimple protocol for ESE1 (ELF3), FRA1 (FOSL1) and NRF2 (NFE2L2) knockdown after 72 hours of treatment with either 0.1% DMSO or 1nM Paclitaxel. **S4C)** Quantification of the images above using default parameters with included Compass software. Total signal indicates the sum of peak area for the +/- 15% range around the highest intensity peak in the +DMSO/-siRNA condition.

Supplementary Figure 5: S5A) HDHB-mClover reporter intensities plot colored by manual assignment. 250 images of cells were randomly selected and manually assigned a cell cycle state (G1, M, S/G2) based on cell morphology and mClover intensity. The manual assignment was used to select Total Intensity Ratio (Nuclear vs cytoplasmic) and Mean intensity ratio (Perinuclear versus Nuclear) as defining features for automatic cell cycle assignment. Black lines represent thresholds used for automated cell cycle assignment. **S5B)** Heatmap showing the heterotypic (between different states) transition rates learned by the Markov model for each unique siRNA +/- Paclitaxel (PTX) condition. Inset number is the transition rate and color is the z-score of row. **S5C, S5D)** Cell count over time plots for each of the DMSO (S6C) and PTX (S6D) treated conditions showing the experimental data (black dots) and Markov values (red line) predicted using the learned transition rates and initial time point. **S5E)** Inferred mitotic failure rate (resulting in multinucleated cells) computed from Markov transition rates.

Supplementary Figure 6: S6A) Representative plot showing the smoothed experimental counts (5-timepoint rolling mean) versus a Loess fit for the siNonTarget + Paclitaxel condition. **S6B)** Dot plots showing the Root Mean Squared Relative Error (RMSRE) for the Markov Model (black dots) over each training epoch versus the Loess fit (green line). Loess fit represents an estimate of the 'noise floor' of the measurement. **S6C)** Model rejection rate for each condition computed from the Chi-squared test applied between the experimental and model predicted composition for each single time point. A nominal Chi-squared p value < 0.05 was considered a significantly different timepoint. A rejection rate of 0% means that there was no significant difference in phenotype composition between observed composition and Markov predicted composition at any timepoint.

Supplementary Figure 7: S7A) Volcano plot showing differentially expressed genes for the Metabric ELF3 high group versus ELF3 low group. Genes to the right (positive Log2FC) are significantly upregulated in the ELF3 high group and genes to the left (negative Log2FC) are significantly upregulated in the ELF3 low group.

Supplemental Figure 1

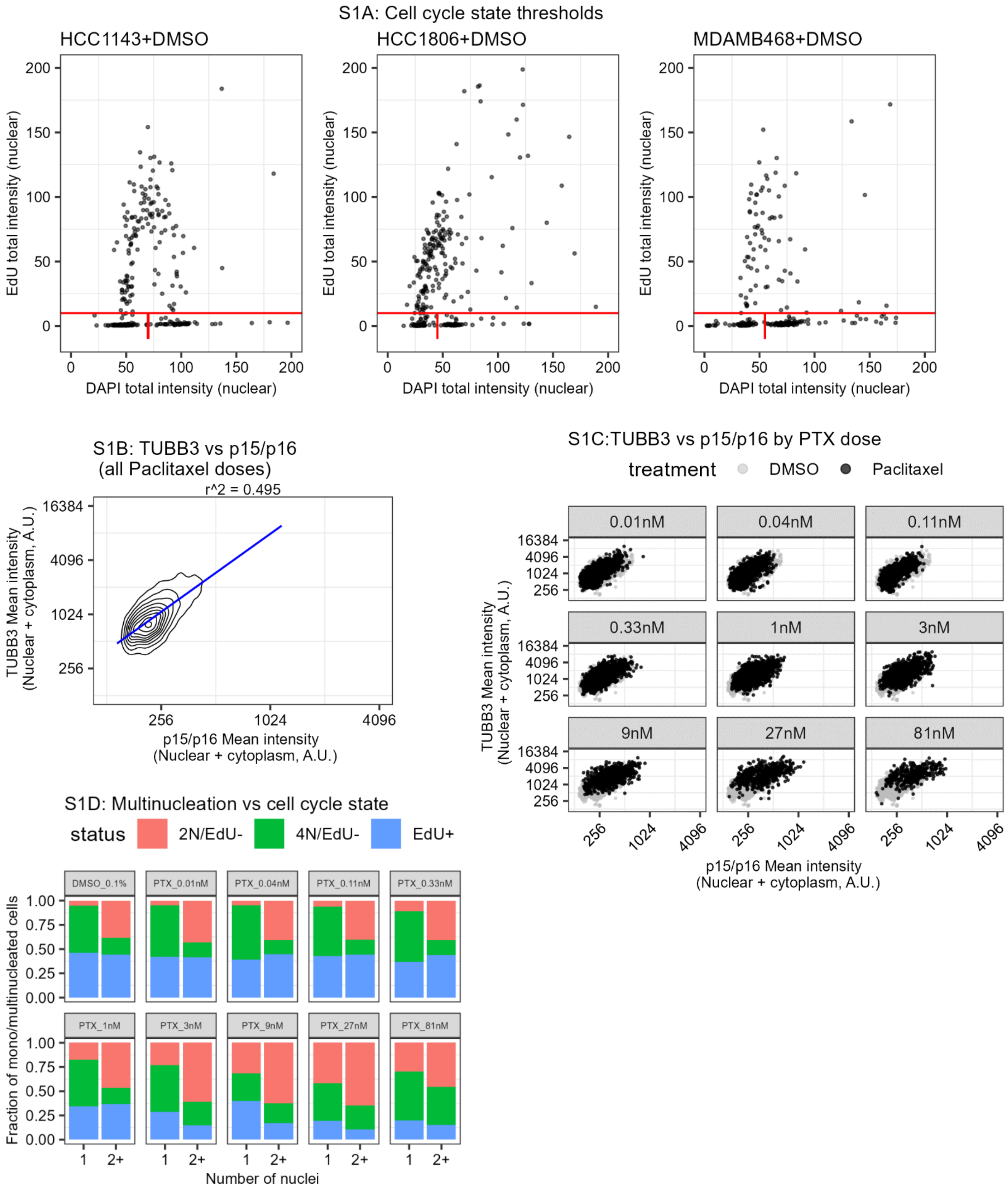

Supplemental Figure 2

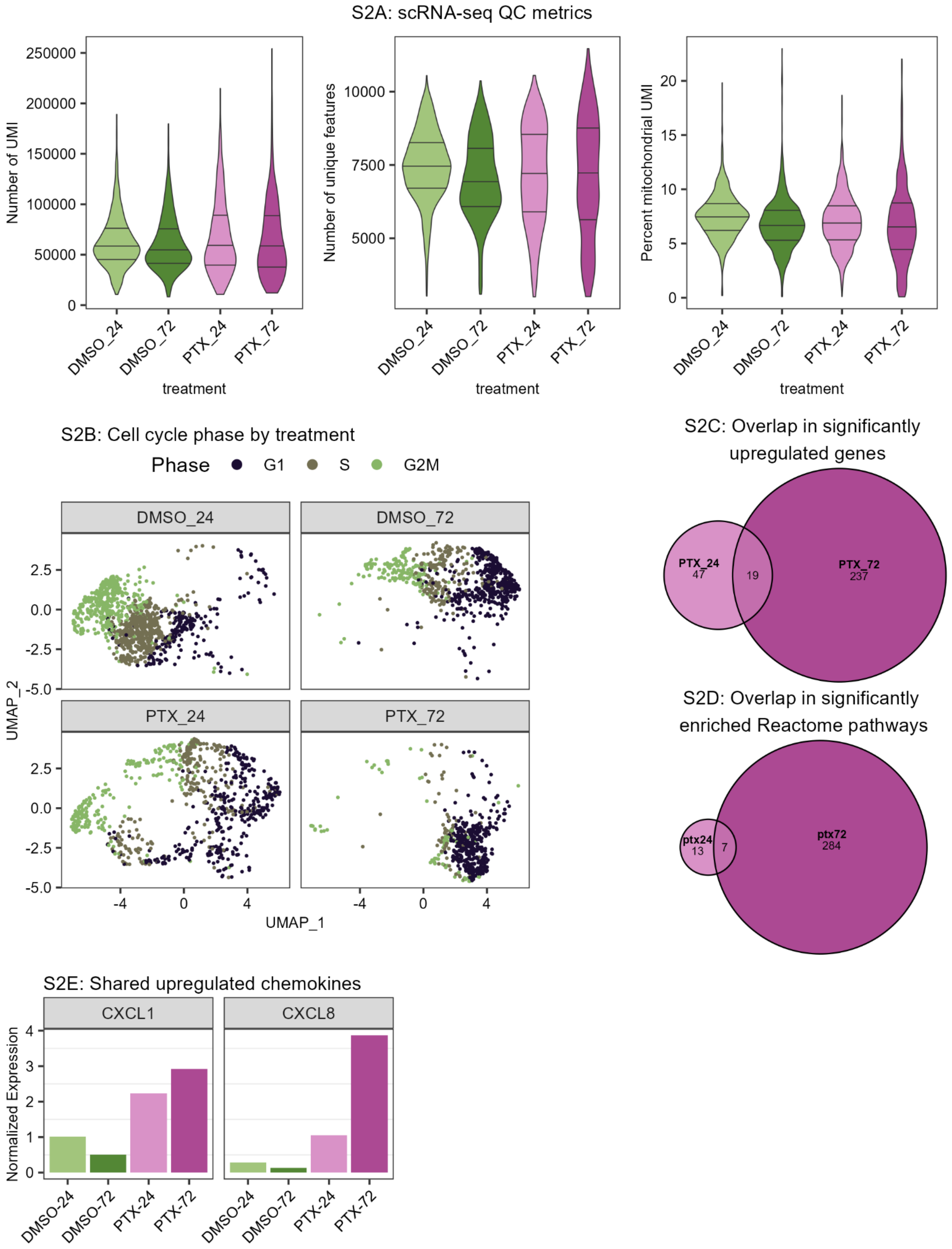

Supplemental Figure 3

S3A: Interferon gene expression

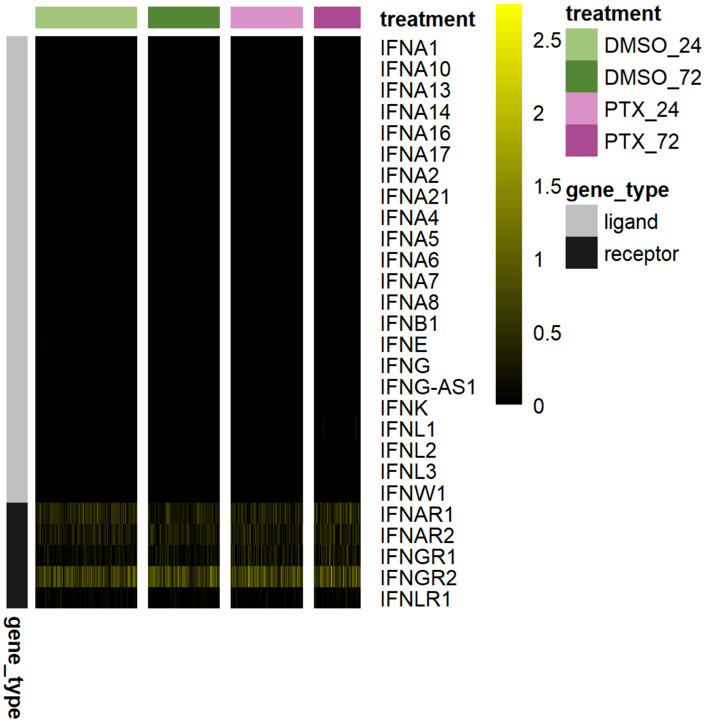

S3B: QC metrics

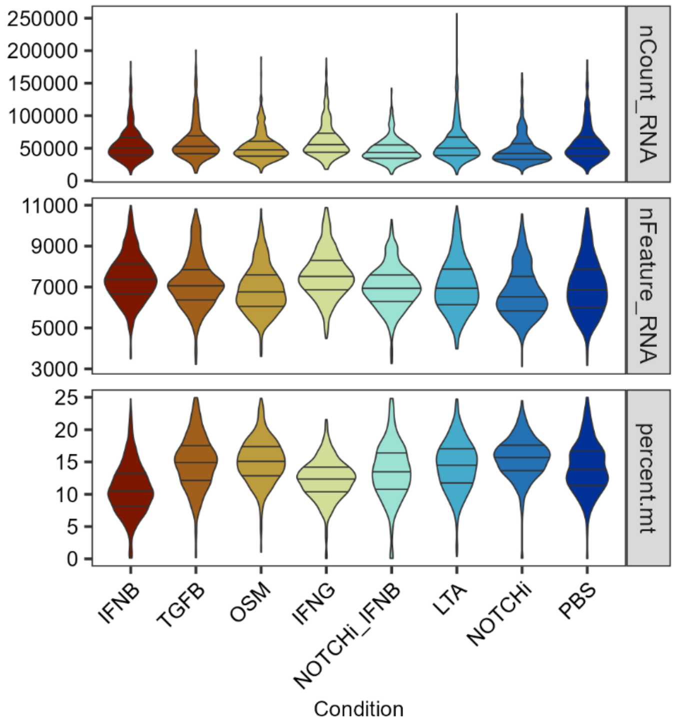

S3D: Cell cycle assignment

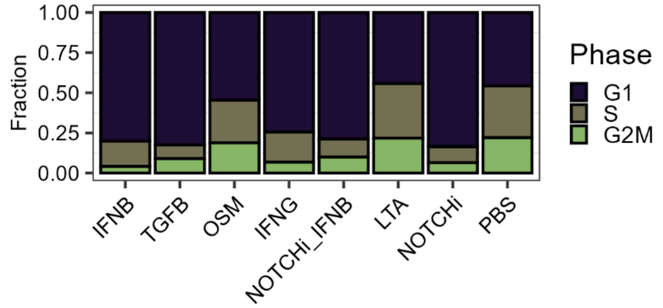

S3C: UMAP by Cell Cycle Phase

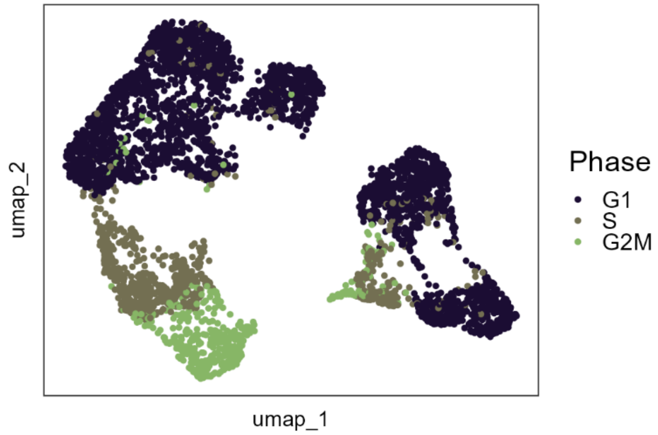

S3F: Single-cell expression correlation
(paclitaxel treated)

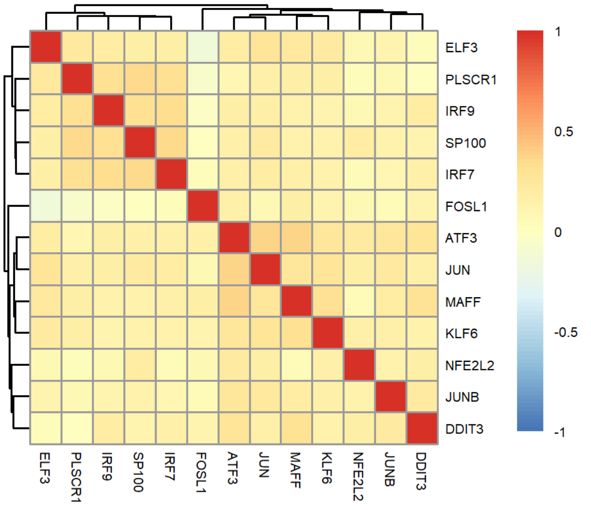

S3E: Interferon does not alter multinucleation

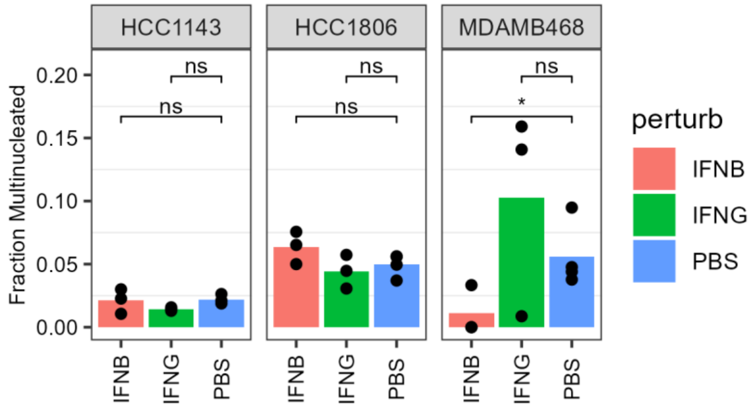

Supplemental Figure 4

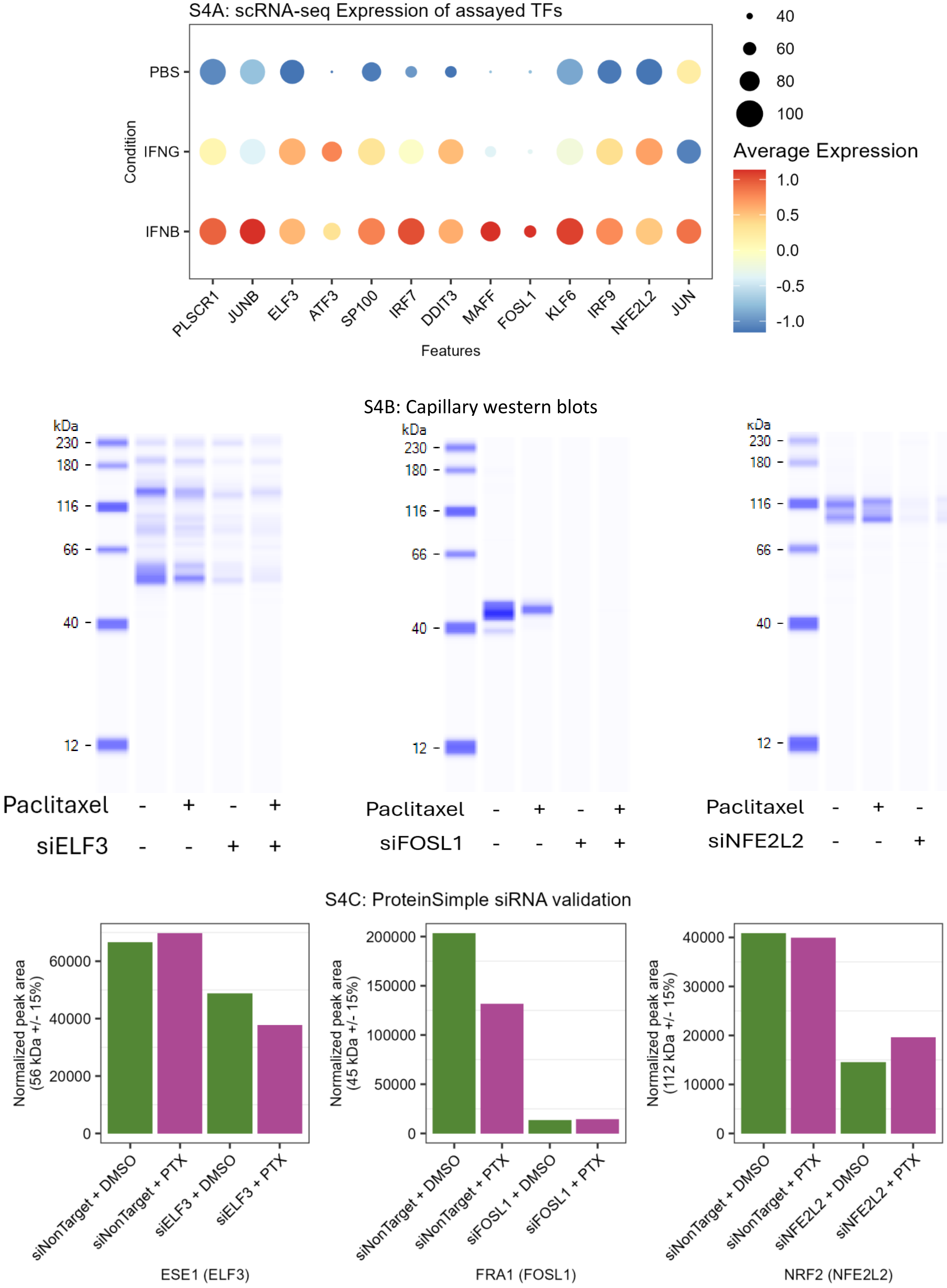

Supplemental Figure 5

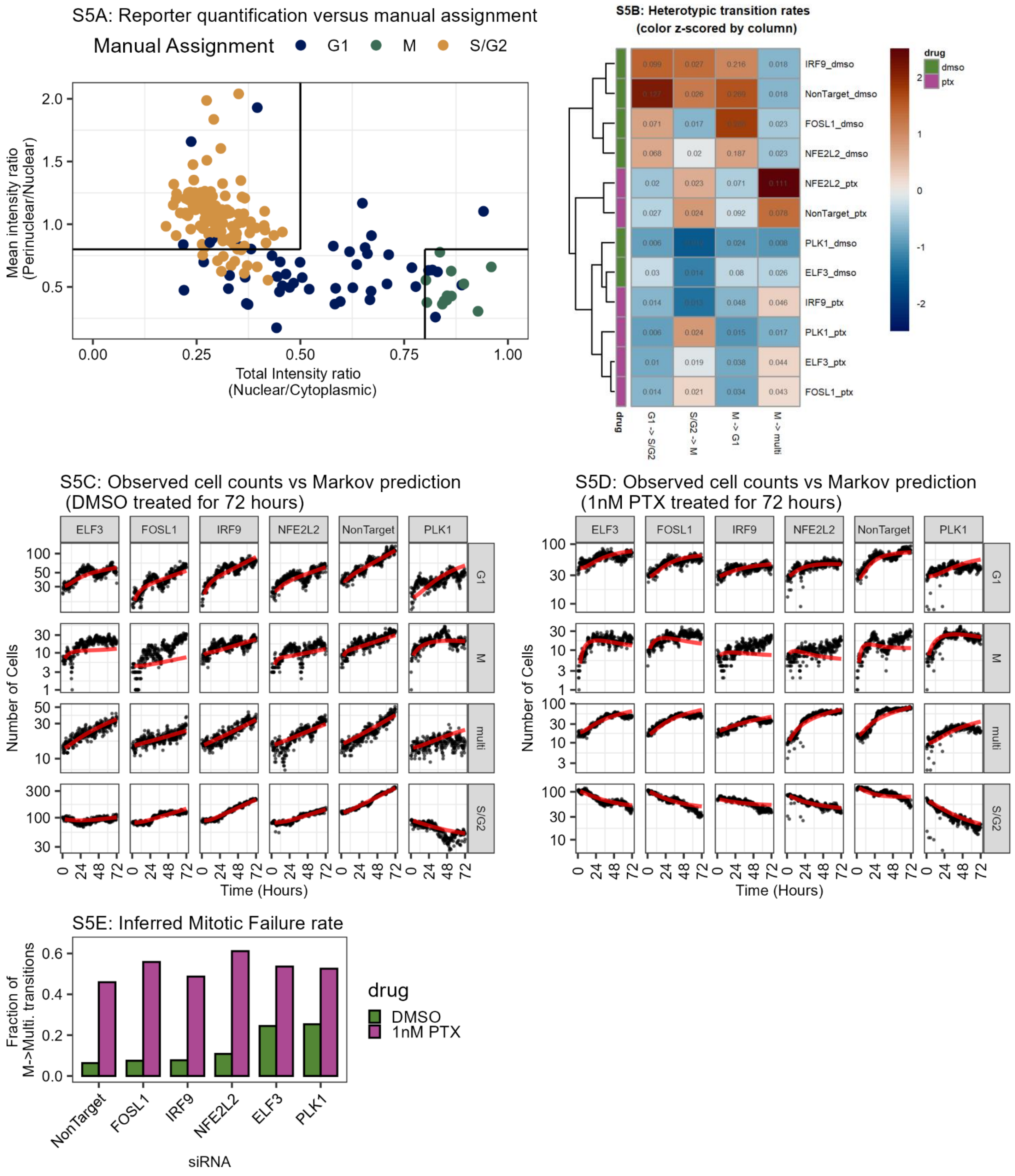

Supplemental Figure 6

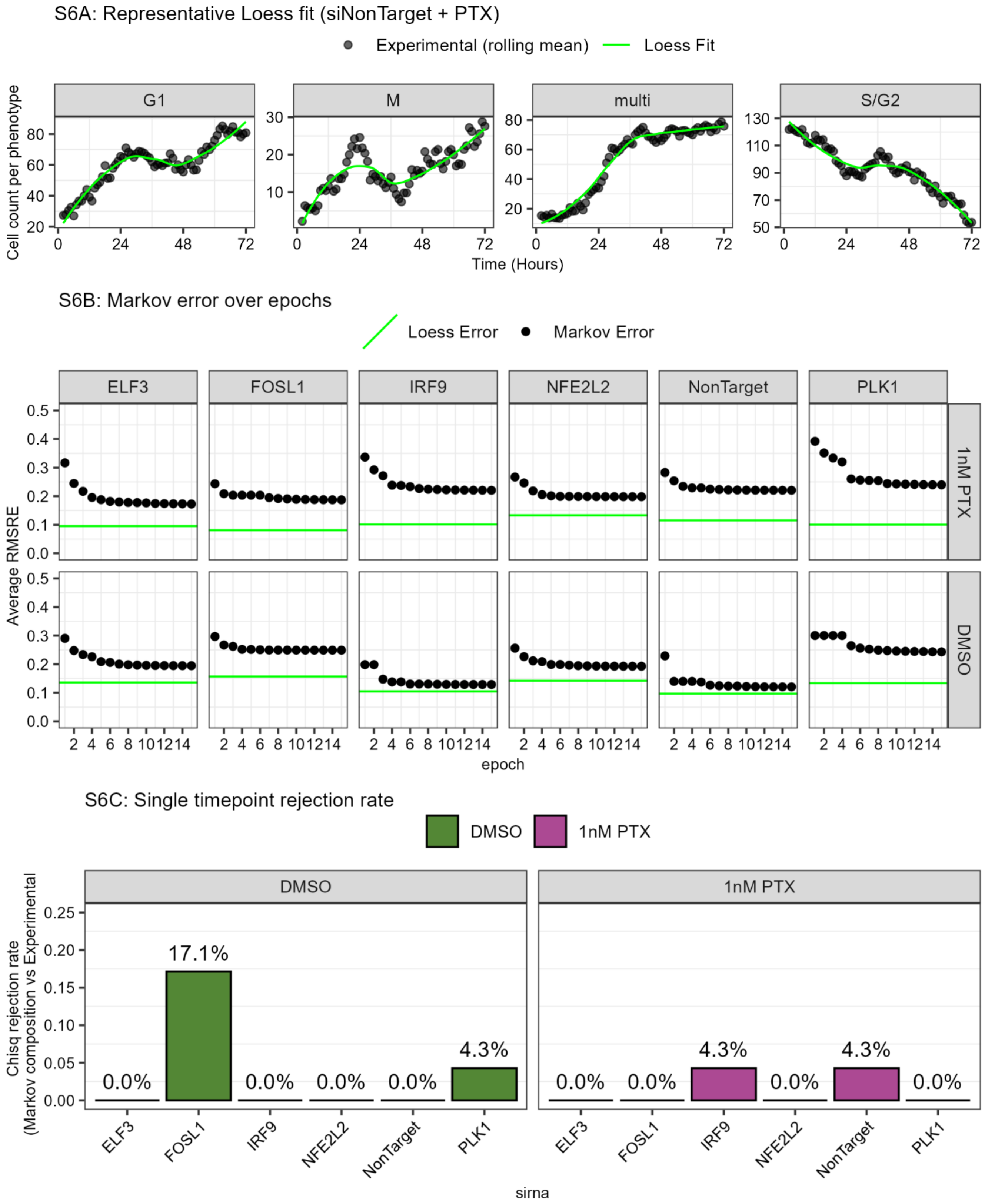

Supplemental Figure 7

S7A: Differential Expression: ELF3 high vs. ELF3 Low

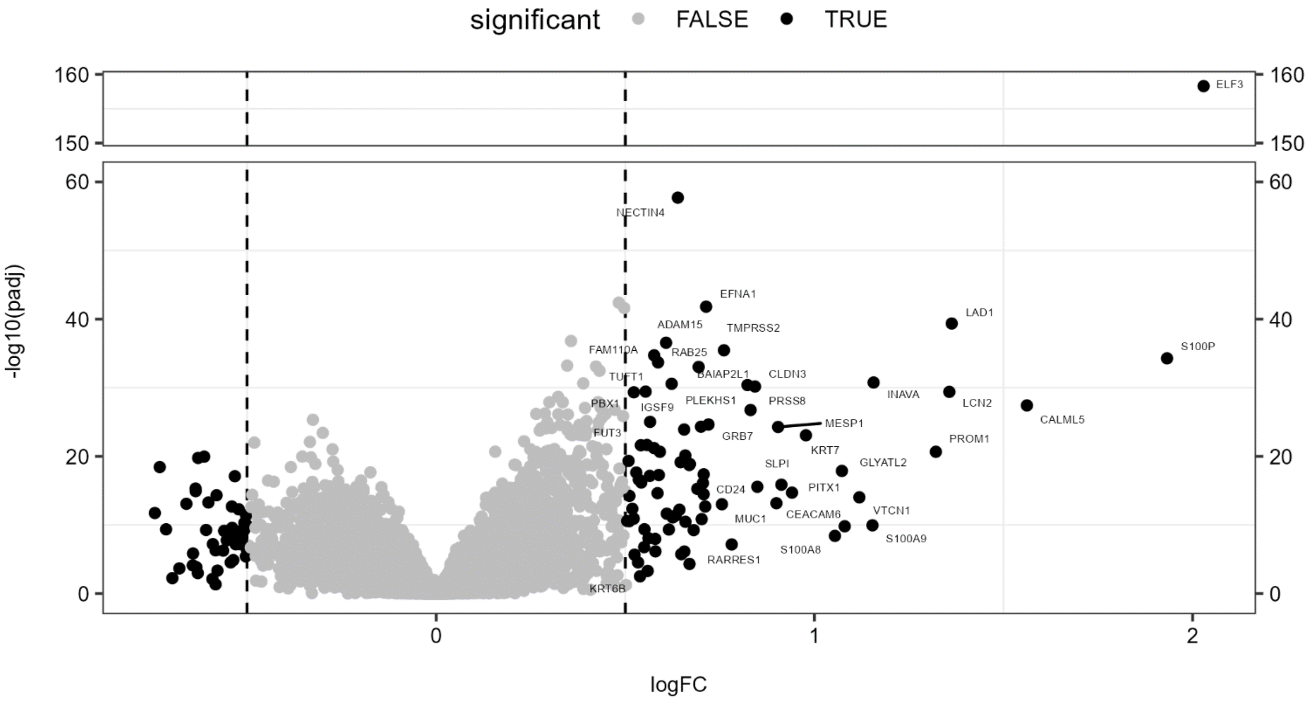

Supplement: Supplementary file 1 — Supplementary Information 1. [file 41598_2024_82218_MOESM1_ESM.pdf]
